# Time–Energy and Time–Entropy Uncertainty Relations in Nonequilibrium Quantum Thermodynamics under Steepest-Entropy-Ascent Nonlinear Master Equations

**DOI:** 10.3390/e21070679

**Published:** 2019-07-11

**Authors:** Gian Paolo Beretta

**Affiliations:** Department of Mechanical and Industrial Engineering, Università di Brescia, via Branze 38, 25123 Brescia, Italy; gianpaolo.beretta@unibs.it

**Keywords:** uncertainty relations, maximum entropy production, steepest-entropy-ascent, quantum thermodynamics, second law of thermodynamics, entropy, nonequilibrium, Massieu

## Abstract

In the domain of nondissipative unitary Hamiltonian dynamics, the well-known Mandelstam–Tamm–Messiah time–energy uncertainty relation $\tau_{F}\Delta_{H}\geq \hbar /2$ provides a general lower bound to the characteristic time $\tau_{F}=\Delta_{F}/\left|d\langle F\rangle /dt\right|$ with which the mean value of a generic quantum observable *F* can change with respect to the width $\Delta_{F}$ of its uncertainty distribution (square root of *F* fluctuations). A useful practical consequence is that in unitary dynamics the states with longer lifetimes are those with smaller energy uncertainty $\Delta_{H}$ (square root of energy fluctuations). Here we show that when unitary evolution is complemented with a steepest-entropy-ascent model of dissipation, the resulting nonlinear master equation entails that these lower bounds get modified and depend also on the entropy uncertainty $\Delta_{S}$ (square root of entropy fluctuations). For example, we obtain the time–energy-and–time–entropy uncertainty relation ${\left(2\tau_{F}\Delta_{H}/\hbar \right)}^{2}+{\left(\tau_{F}\Delta_{S}/k_{B}\tau \right)}^{2}\geq 1$ where τ is a characteristic dissipation time functional that for each given state defines the strength of the nonunitary, steepest-entropy-ascent part of the assumed master equation. For purely dissipative dynamics this reduces to the time–entropy uncertainty relation $\tau_{F}\Delta_{S}\geq k_{B}\tau$, meaning that the nonequilibrium dissipative states with longer lifetime are those with smaller entropy uncertainty $\Delta_{S}$.

## 1. Introduction

Recent advances in quantum information and quantum thermodynamics (QT) have increased the importance of estimating the lifetime of a given quantum state, for example to engineer decoherence correction protocols aimed at entanglement preservation. In the same spirit as fluctuation theorems that allow to estimate some statistical features of the dynamics from suitable state properties, also the Mandelstam–Tamm–Messiah time–energy uncertainty relations (MTM-TEURs) have been long known to provide bounds on lifetimes of quantum decaying states under Hamiltonian (non-dissipative) evolution. For practical applications, however, such bounds are insufficient when Hamiltonian dynamics must be complemented by models of dissipation and decoherence.

The time–energy uncertainty relation has remained an open and at times controversial issue throughout the history of quantum theory. Several reviews are available on the pioneering discussions and the subsequent developments [1,2,3,4,5,6,7,8,9,10,11,12,13,14,15]. In the present paper, we are motivated by the past two decades of important advancements in our understanding of the general structure of dynamical models for non-equilibrium thermodynamics, including non-equilibrium quantum thermodynamic models. Such revival has been prompted and paralleled by a steady advancement of experimental techniques dealing with single ion traps [16,17], qubits [18,19], neutron interferometry [20,21], and a countless and growing number of other quantum-information developments since then, e.g., nonlinear quantum metrology [22,23]. Within these applications, TEURs can provide useful information and practical bounds for parameter estimation. But since dissipation and decoherence are often the limiting factors, there is a need to generalize the MTM-TEURs to frameworks where microscopic few-particle quantum setups exhibit non-unitary dissipative dynamical behavior.

A recent review paper on the physical significance of TEURs provides 300 references and the following conclusion [24]: “We have shown that the area of energy–time uncertainty relations continues to attract attention of many researchers until now, and it remains alive almost 90 years after its birth. It received a new breath in the past quarter of century due to the actual problems of quantum information theory and impressive progress of the experimental technique in quantum optics and atomic physics. It is impossible to describe various applications of the TEURs to numerous different physical phenomena in this minireview.”

The main objective in the present paper is to extend the time–energy uncertainty relations to the framework of dissipative quantum dynamical systems. But differently from the most popular and traditional model of dissipation in open quantum systems, which is based on the well-known Kossakowski–Lindblad–Gorini–Sudarshan (KLGS) master equations [25,26,27,28,29,30,31,32,33], we assume the less known locally steepest-entropy-ascent (LSEA) model of dissipation. We make this choice not only to avoid some drawbacks (outlined in more details in Appendix A) of the KLGS master equation from the point of view of full and strong consistency with the general principles of thermodynamics, causality, and far non-equilibrium, but more importantly because we have shown in References [34,35] that the LSEA principle—by providing the minimal but essential elements of thermodynamic consistency, near as well as far from stable (maximal entropy) equilibrium states—has the potential to unify all the successful frameworks of non-equilibrium modeling, from kinetic theory to chemical kinetics, from stochastic to mesoscopic to extended irreversible thermodynamics, as well as the metriplectic structure or, in more recent terms, the General Equation for Non-Equilibrium Reversible-Irreversible Coupling (GENERIC) structure. In addition, it is noteworthy that a particular but broad class of KLGS master equations has been recently shown to fall into a LSEA (entropic gradient flow) structure [36,37], and hence some of the TEURs we derive here hold also for such class of models.

Steepest-entropy-ascent (SEA) nonlinear master equations have proved to be effective tools to model dissipative dynamics, thermalization, transport processes and, in general, entropy production in a wide range of frameworks of non-equilibrium thermodynamics. In essence, SEA models are explicit implementations of the general principle of maximum local entropy production. In recent mathematical terms, SEA models are entropic gradient flows. From the fundamental point of view, the general structure and nonlinearity of the SEA master equations are instrumental to providing strong compatibility with the second law of thermodynamics by guaranteeing, within the model, the Hatsopoulos–Keenan statement of existence and uniqueness of the stable equilibrium (maximum entropy) states. Here we focus on the quantum thermodynamic modeling framework of application and show how the entropy production modifies the usual TEURs.

The usual time–energy uncertainty relation—as interpreted according to the Mandelstam–Tamm– Messiah intrinsic-time approach [38,39] based on unitary Hamiltonian dynamics—is modified by the presence of a maximally dissipative term in the dynamical law, which models at the single- or few-particle quantum level the so-called maximum entropy production principle (MEPP) [40,41,42,43,44,45,46,47]. TEURs obtained in other frameworks [48,49,50,51,52,53,54,55,56] such as attempts to define time or “tempus” operators, entropic uncertainties, and measurement times are beyond our scope here.

The class of MEPP master equations we designed in References [57,58,59,60,61,62] is suitable to model dissipation phenomenologically not only in open quantum systems in contact with macroscopic baths, but also in closed isolated systems, as well as strongly coupled and entangled composite systems (references below). These master equations are capable to describe the natural tendency of any initial nonequilibrium state (read: density operator) to relax towards canonical or partially-canonical thermodynamic equilibrium (Gibbs state), i.e., capable of describing the irreversible tendency to evolve towards the highest entropy state compatible with the instantaneous mean values of the energy (and possibly other constants of the motion and other constraints). They do so by preserving exactly the conserved properties while pulling every nonequilibrium state in the SEA direction with respect to the local dissipation metric that is part of the nonequilibrium description of the system [34]. This dissipative tendency is simultaneous and in competition with the usual non-dissipative Hamiltonian unitary evolution.

Our original approach—when understood as an attempt to develop thermodynamically consistent modeling approaches that merge mechanics and thermodynamics following Hatsopoulos and Gyftopoulos [63,64,65,66]—can perhaps be considered a first pioneering “resource theory” of quantum thermodynamics equipped with a nonlinear dissipative dynamical structure capable to describe relaxation even from arbitrarily far from equilibrium and to entail the second law as a theorem of the dynamical law. Several other pioneering aspects of QT resource theories were present in References [63,64,65,66]. For example, the energy versus entropy diagram to represent nonequilibrium states in the QT framework, first introduced in Reference [65], has recently found interesting applications in [67]. Again, it provides definitions and expressions for adiabatic availability and available energy with respect to a heat bath, work element, heat interaction, etc. which are currently discussed intensely in the QT community. It must also be mentioned that this first QT resource theory was proposed in years when talking of quantum thermodynamics was considered heresy by the orthodox physical community. Considering that it is little cited and still not well known, we give more details below and in Appendix B.

We provide in the two appendices a brief review of some practical and conceptual issues of the prevailing model of irreversibility, and a discussion of the original motivation that lead us to develop a quantum maximal entropy production formalism. We do not repeat here the geometrical derivations of our nonlinear MEPP dynamical law, nor the discussions of its many intriguing mathematical–physics implications, because they are available in many previous papers. Here, we simply adopt that master equation without derivation, and focus on its consequences related to TEURs, illustrated also by some numerical simulations. Quantum statistical mechanics and quantum thermodynamics practitioners have so far essentially dismissed and ignored our class of SEA master equations on the basis that they do not belong to the standard class of KLGS master equations and hence cannot be the correct description of the reduced dynamics of a system in interaction with one or more thermal baths. However, at least when used as phenomenological modeling tools, SEA master equations have recently proved [68,69,70,71,72,73,74,75,76] to offer in a variety of fields important advantages of broader or complementary applicability for the description and correlation of near- and far-non-equilibrium behavior.

In the quantum framework, the local state of a subsystem is represented by the local density operator ρ and its lifetime may be characterized by the intrinsic characteristic times $\tau_{F}$ of the dynamical variables associated with the linear functionals Tr(ρF). If the local dynamics is non-dissipative and described by the usual unitary evolution, we show below that the Heisenberg–Robertson inequality entails the usual MTM-TEURs $\tau_{F}\Delta_{H}\geq \hbar /2$, while the Schroedinger inequality entails sharper and more general exact TEURs [Equation (23)].

For simultaneous unitary+dissipative dynamics, the usual TEUR is expectedly replaced by less restrictive relations and additional characteristic times acquire physical significance. In particular, we focus our attention to the characteristic time associated with the rate of change of the von Neumann entropy functional $-k_{B}\mathrm{Tr}\left(\rho \ln\rho \right)$. For unitary+LSEA evolution, in Section 7 [Equation (83)] we obtain an interesting time–energy and time–entropy uncertainty relation ${\left(2\tau_{F}\Delta_{H}/\hbar \right)}^{2}+{\left(\tau_{F}\Delta_{S}/k_{B}\tau \right)}^{2}\geq 1$ where τ is the main dissipation time that defines the strength of the dissipative component of the assumed dynamical law. With the help of numerical simulations, we illustrate this relation and several other even more precise uncertainty relations, that in the framework of QT resource theories may have a useful application in quantifying the lifetime of quantum states.

The structure of the paper is outlined at the end of the next section, where we first introduce the particular class of nonlinear dissipative quantum master equations on which we restrict our attention in the first part of the paper.

## 2. Assumed Structure of the Nonlinear Dissipative Quantum Master Equation

Let H (dimH≤∞) be the Hilbert space and *H* the Hamiltonian operator that in standard Quantum Mechanics we associate with a given isolated (or adiabatic, see below) and uncorrelated system. We assume that the quantum states are one-to-one with the linear hermitian operators ρ on H with Tr(ρ)=1 and $\rho \geq \rho^{2}$, and we assume a dynamical equation of the form

(1) $$\frac{d\rho }{dt}=\rho \;E\left(\rho \right)+E^{†}\left(\rho \right)\;\rho \;,$$

where E(ρ) is an operator-valued function of ρ that we may call the “evolution generator” which may in general be non-hermitian and nonlinear in ρ, but must be such as to preserve ρ unit trace and non-negative definite. Without loss of generality, we write $E=E_{+}+iE_{-}$ where $E_{+}=\left(E+E^{†}\right)/2$ and $E_{-}=\left(E-E^{†}\right)/2i$ are hermitian operators. Then, the dynamical law takes the form

(2) $$\frac{d\rho }{dt}=-i\;\left[E_{-},\rho \right]+\left\{E_{+},\rho \right\}\;,$$

where [·,·] and {·,·} are the usual commutator and anticommutator, respectively. In Appendix A we discuss the reasons why we adopt this form, and exclude terms like V(ρ)ρV(ρ) which appear instead in the the celebrated KSGL class of (linear) quantum master equations.

In preparation for our SEA construction in Section 7, we assume $E_{-}=H/\hbar$ (independent of ρ), where *H* is the Hamiltonian operator of the system and *ℏ* the reduced Planck constant, and rewrite $E_{+}$ as $E_{+}=\Delta M\left(\rho \right)/2k_{B}\tau$ where $k_{B}$ is the Boltzmann constant, τ a positive constant (or state functional) that in the SEA framework we will interpret as an intrinsic dissipation time of the system, because it essentially fixes the rate at which the state evolves along the path of SEA in state space, and ΔM(ρ) a hermitian operator-valued nonlinear function of ρ that we call the “nonequilibrium Massieu operator” and until Section 7 we do not define explicitly, except for the assumption that it satisfies the condition

(3) Tr[ρΔM(ρ)]=0

as well as the condition that it preserves the nonnegativity of ρ (both forward and backwards in time!). As a result, Equation (1) takes the form

(4) $$\frac{d\rho }{dt}=-\frac{i}{\hbar }\left[H,\rho \right]+\frac{1}{2k_{B}\tau }\left\{\Delta M\left(\rho \right),\rho \right\}\;.$$

Let us note that as in standard unitary dynamics, we say that the particle is either isolated or adiabatic, respectively, if the Hamiltonian operator *H* is either time independent or time dependent, for example, through one or more external control parameters.

In Section 7, we will consider for ΔM(ρ) the explicit SEA form for the simplest case, first proposed in [57,58,61]. Reference [59] proposed also a general LSEA form for a composite quantum system, which will not be considered here, but has clear applications in the description of decoherence and lifetime of entanglement (see Reference [68]).

In the present paper we limit the discussion to the derivation of general inequalities, and to illustrative considerations and a numerical example valid within the simplest framework of steepest-entropy-ascent conservative dynamics. The application to structured composite systems based on our LSEA version [59,68] of operator ΔM(ρ) will be discussed elsewhere.

The specific physical interpretations of the uncertainty relations that follow from dynamical law (4) will depend on the theoretical or modeling context in which such time evolution is assumed. For example, the problem of designing well-behaved nonlinear extensions of the standard unitary dynamical law of quantum mechanics has been faced in the past few decades with a variety of motivations, and is recently seeing a vigorous revival in connection with questions about the foundations of quantum mechanics and the need for thermodynamically sound phenomenological models (recently referred to as “resource theories” [67]) that arise from the current developments of quantum information technologies and related single-particle and single-photon experiments to test quantum computing components and devices and fundamental questions about entanglement, decoherence, nonlocality, and measurement theory.

In our original development [57,58,59,60,61], Equation (4) was designed as part of an ad-hoc fundamental dynamical postulate needed [77,78,79,80,81,82] to complete the Hatsopoulos-Gyftopoulos attempt [63,64,65,66,83,84] to unify mechanics and thermodynamics into a generalized quantum theory by building the Hatsopoulos–Keenan statement of the second law [85,86] directly into the microscopic level of description. In particular, the key ansatz in References [63,64,65,66] is the assumption that even for a strictly isolated system, there exists a broad class of genuine states (homogeneous preparations, in von Neumann language [57,82,87,88]) that require non-idempotent density operators, i.e., such that $\rho^{2}\neq \rho$. Two decades later, this ansatz has been re-proposed in Reference [89], and our nonlinear dynamical Equation (4) has been re-discovered and studied in References [90,91,92,93], where it is shown to be well-behaved from various perspectives including a relativistic point of view. An important feature is that it entails non-unitary evolution only for non-idempotent ($\rho^{2}\neq \rho$) density operators, whereas for idempotent ($\rho^{2}=\rho$) density operators it entails the standard unitary evolution (see, e.g., References [58,94]).

However, the present results are valid also in any other framework, theoretical discussion, modeling context, or resource theory whereby—for example to study decoherence, dissipation, quantum thermal engines, quantum refrigerators, and so on—the usual Liouville-von Neumann equation for the density operator is modified, linearly or nonlinearly, into form (4).

Since many of the relations we derive here are valid and nontrivial in all these contexts, in Section 3, Section 4, Section 5 and Section 6 we begin by presenting the results that do not depend on assuming a particular form of operator ΔM(ρ). Thus, independently of the interpretation, the context of application, and the specific form of master Equation (4), the uncertainty relations derived in the first part of the paper extend the usual relations to the far non-equilibrium domain and in general to all non-zero-entropy states.

In Section 7 and Section 8, to fix ideas and be able to present numerical results and qualitative considerations, we specialize the analysis to the simplest nontrivial form of Equation (4) that implements our conservative steepest-entropy-ascent dynamical ansatz, namely, a model for irreversible relaxation of a four-level qudit.

Appendix A discusses our reasons for not considering, in the present context, the extension of our results to a full Kossakowski-Lindblad form of the evolution equation.

Appendix B gives a brief review of the original motivations that lead us to develop the SEA and LSEA formalism in the early quantum thermodynamics scenario, and of the subsequent developments that in recent years have shown how the locally steepest-entropy-ascent principle not only gives a clear, explicit, and unambiguous meaning to the MEPP but it also constitutes the heart of (and essentially unifies) all successful theories of nonequilibrium.

## 3. General Uncertainty Relations

We consider the space L(H) of linear operators on H equipped with the real scalar product

(5) $$\left(F|G\right)=\mathrm{Tr}\left(F^{†}G+G^{†}F\right)/2=\left(G|F\right)\;,$$

and the real antisymmetric bilinear form

(6) $$\left(F\backslash G\right)=i\;\mathrm{Tr}\left(F^{†}G-G^{†}F\right)/2=-\left(G\backslash F\right)=\left(F|iG\right)\;,$$

so that for any hermitian *F* in L(H) the corresponding mean-value state functional can be written as $\langle F\rangle =\mathrm{Tr}\left(\rho F\right)=\mathrm{Tr}\left(\sqrt{\rho }F\sqrt{\rho }\right)=\left(\sqrt{\rho }|\sqrt{\rho }F\right)$, and can therefore be viewed as a functional of $\sqrt{\rho }$, the square-root density operator, obtained from the spectral expansion of ρ by substituting its eigenvalues with their positive square roots. When ρ evolves according to Equation (1) and *F* is time-independent, the rate of change of Tr(ρF) can be written as

(7) $$d\mathrm{Tr}\left(\rho F\right)/dt=\mathrm{Tr}\left(F\;d\rho /dt\right)=2\left.\left(\sqrt{\rho }F\right|\sqrt{\rho }E\left(\rho \right)\right)\;.$$

In particular, for the evolution Equation (1) to be well defined, the functional Tr(ρI) where *I* is the identity on H must remain equal to unity at all times; therefore, $d\mathrm{Tr}\left(\rho I\right)/dt=2\left.\left(\sqrt{\rho }I\right|\sqrt{\rho }E\left(\rho \right)\right)=0$ or, equivalently, in view of Equation (4), Equation (3) rewrites as

(8) $$\left.\left(\sqrt{\rho }\right|\sqrt{\rho }\Delta M\left(\rho \right)\right)=0\;.$$

For *F* and *G* hermitian in L(H), we introduce the following shorthand notation

(9) $$\begin{array}{ccc} \Delta F & = & F-\mathrm{Tr}(\rho F)I\;, \end{array}$$

(10) $$\begin{array}{ccc} \sigma_{\;F\;G} & = & \langle \Delta F\Delta G\rangle =(\sqrt{\rho }\Delta F|\sqrt{\rho }\Delta G)\\ & = & \frac{1}{2}\mathrm{Tr}\left(\rho \left\{\Delta F,\Delta G\right\}\right)=\sigma_{\;G\;F}\;, \end{array}$$

(11) $$\begin{array}{ccc} \Delta_{F} & = & \sqrt{\sigma_{\;F\;F}}=\sqrt{\langle \Delta F\Delta F\rangle }\;, \end{array}$$

(12) $$\begin{array}{ccc} \eta_{F\;G} & = & \langle [F,G]/2i\rangle =(\sqrt{\rho }\Delta F\backslash \sqrt{\rho }\Delta G)\\ & = & \frac{1}{2i}\mathrm{Tr}\left(\rho \left[F,G\right]\right)=\eta_{F\;G}^{*}=-\eta_{G\;F}\;, \end{array}$$

For example, we may write the rate of change of the mean value of a time-independent observable *F* as

(13) $$\frac{d\mathrm{Tr}(\rho F)}{dt}=\frac{\langle [F,H]/2i\rangle }{\hbar /2}+\frac{\langle \Delta F\Delta M\rangle }{k_{B}\tau }=\frac{\eta_{F\;H}}{\hbar /2}+\frac{\sigma_{\;F\;M}}{k_{B}\tau }\;,$$

from which we see that not all operators *F* that commute with *H* correspond to constants of the motion, but only those for which 〈ΔFΔM〉=0, i.e., such that $\sqrt{\rho }\Delta F$ is orthogonal to both $i\sqrt{\rho }\Delta H$ and $\sqrt{\rho }\Delta M$, in the sense of scalar product (5). For an isolated system, conservation of the mean energy functional Tr(ρH) requires an operator function ΔM(ρ) that maintains $\sqrt{\rho }\Delta M$ always orthogonal to $\sqrt{\rho }\Delta H$, so that 〈ΔHΔM〉=0 for every ρ.

From the Schwarz inequality, we readily verify the following generalized Schrödinger uncertainty relation

(14) $$\langle \Delta F\Delta F\rangle \langle \Delta G\Delta G\rangle \geq {\langle \Delta F\Delta G\rangle }^{2}+{\langle [F,G]/2i\rangle }^{2}\;,$$

usually written in the form $\sqrt{\sigma_{\;F\;F}\sigma_{\;G\;G}-\sigma_{\;F\;G}^{2}}\geq \left|\eta_{F\;G}\right|$. It follows from the Cauchy–Schwarz inequality $\left(f,f\right)\left(g,g\right)\geq {\left|\left(f,g\right)\right|}^{2}$ and the identity ${\left|\left(f,g\right)\right|}^{2}={\left(f|g\right)}^{2}+{\left(f\backslash g\right)}^{2}$ where (f|g)=[(f,g)+(g,f)]/2, (f\g)=i[(f,g)−(g,f)]/2, and *f*, *g* are vectors in some complex Hilbert space (strict equality iff f=λg for some scalar λ). In the space $L^{\prime }\left(H\right)$ of linear operators on H equipped with the complex scalar product $\left(f,g\right)=\mathrm{Tr}\left(f^{†}g\right)$, we note that (f,f)=(f|f) and obtain the inequality $\left(f|f\right)\left(g|g\right)\geq {\left(f|g\right)}^{2}+{\left(f\backslash g\right)}^{2}$ and hence inequality (14) by setting $f=\sqrt{\rho }\Delta F$ and $g=\sqrt{\rho }\Delta G$. Note that the strict equality in (14) holds iff $\sqrt{\rho }\Delta F=\lambda \sqrt{\rho }\Delta G$ for some scalar λ (in which case we have 〈[F,G]/2i〉=0 iff either $\lambda^{*}=\lambda$ or $\sqrt{\rho }\Delta F=0$ or both). This proof was given as footnote 7 of Reference [95]. For Schroedinger’s original proof and an alternative one see Reference [96]. Relation (14) is a generalization of the inequality first appeared in [97,98] and later generalized in [99] to the form $\det\sigma =\sigma_{\;F\;F}\sigma_{\;G\;G}-\sigma_{\;F\;G}^{2}\geq \det\eta =\eta_{F\;G}^{2}=\eta_{G\;F}^{2}$, suitable for generalizations to more than two observables. Early proofs of relation (14) were restricted to pure state operators ($\rho^{2}=\rho$). To our knowledge, the earliest proof valid for general (mixed and pure) states ρ is that in [6]. For further inequalities in the case of position and momentum operators see [14] and references therein. Notice also that by using our proof of the Schrodinger inequality (14), just given above, Relation (22) of the main theorem in the review paper [15] can be made sharper and read ${\left|\mathrm{Tr}\left(R\left[A,B\right]\right)\right|}^{2}+{\left|\mathrm{Tr}\left(R\left\{A,B\right\}\right)-2\sum_{n}\lambda_{n}\mathrm{Tr}\left(P_{n}AP_{n}B\right)\right|}^{2}\leq 4f^{2}\left(R,A\right)f^{2}\left(R,B\right)$.

Relation (14) obviously entails the less precise and less symmetric Heisenberg-Robertson uncertainty relation

(15) $$\langle \Delta F\Delta F\rangle \langle \Delta G\Delta G\rangle \geq {\langle [F,G]/2i\rangle }^{2}\;,$$

usually written in the form $\Delta_{F}\Delta_{G}\geq \left|\eta_{F\;G}\right|$.

For further compactness, we introduce the notation

(16) $$\begin{array}{ccc} r_{FG} & = & \sigma_{\;F\;G}/\sqrt{\sigma_{\;F\;F}\sigma_{\;G\;G}}\;,\\ c_{FG} & = & \eta_{F\;G}/\sqrt{\sigma_{\;F\;F}\sigma_{\;G\;G}}\;, \end{array}$$

where clearly, $r_{FG}$ represents the cosine of the angle between the ‘vectors’ $\sqrt{\rho }\Delta F$ and $\sqrt{\rho }\Delta G$ in L(H), and $r_{FG}^{2}\leq 1$. Inequality (14) may thus be rewritten as

(17) $$r_{FG}^{2}+c_{FG}^{2}\leq 1$$

and clearly implies

(18) $$c_{FG}^{2}\leq \frac{1}{1+(r_{FG}^{2}/c_{FG}^{2})}\leq 1-r_{FG}^{2}\leq 1\;.$$

Next, for any hermitian *F* we define the characteristic time of change of the corresponding property defined by the mean value of the linear functional 〈F〉=Tr(ρF) as follows

(19) $$\tau_{F}\left(\rho \right)=\Delta_{F}/\left|d\langle F\rangle /dt\right|\;.$$

As is well known [1,2,3,4,5,7,8,10,11,14,15,38,39,48], $\tau_{F}$ represents the time required for the statistical distribution of measurements of observable *F* to be appreciably modified, i.e., for the mean value 〈F〉 to change by an amount equal to the width $\Delta_{F}$ of the distribution.

Now, defining the nonnegative, dimensionless functional

(20) $$a_{\tau }=\hbar \Delta_{M}/2k_{B}\tau \Delta_{H}\;,$$

we rewrite (13) in the form

(21) $$d\langle F\rangle /dt=2\Delta_{F}\Delta_{H}\;\left(c_{F\;H}+a_{\tau }\;r_{\;F\;M}\right)/\hbar$$

and, substituting into (19), we obtain the general exact uncertainty relation

(22) $$\frac{\hbar /2}{\tau_{F}\Delta_{H}}=\left|c_{F\;H}+a_{\tau }\;r_{\;F\;M}\right|\;.$$

For non-dissipative dynamics [ΔM(ρ)/τ=0], $a_{\tau }=0$, Equation (22) yields the time–energy uncertainty relations

(23) $$\frac{\hbar^{2}/4}{\tau_{F}^{2}\sigma_{\;H\;H}}=c_{\;F\;H}^{2}\leq \frac{1}{1+(r_{\;F\;H}^{2}/c_{\;F\;H}^{2})}\leq 1-r_{\;F\;H}^{2}\leq 1\;,$$

which entail but are more precise than the usual time–energy uncertainty relation, in the same sense as Schrödinger’s relation (14) entails but is more precise than Heisenberg’s relation (15). According to (19), the last inequality in (23) implies that property 〈F〉 cannot change at rates faster than $2\Delta_{F}\Delta_{H}/\hbar$.

For dissipative dynamics let us first consider an observable *A* that commutes with *H*, so that 〈[A,H]/2i〉=0 while 〈ΔAΔH〉≠0; in other words, an observable conserved by the Hamiltonian term in the dynamical law (4), but not conserved by the dissipative term. Then Equation (22) yields the equivalent time–energy uncertainty relations

(24) $$\frac{\hbar /2}{\tau_{A}\Delta_{H}}=a_{\tau }\;\left|r_{\;A\;M}\right|\leq a_{\tau }\;,$$

(25) $$\frac{k_{B}\tau }{\tau_{A}\Delta_{M}}=\left|r_{\;A\;M}\right|\leq 1\;.$$

We note that while $r_{\;A\;M}^{2}\leq 1$, the value of $a_{\tau }$ depends on how ΔM(ρ)/τ is defined and, a priori, could well be larger than unity, in which case there could be some observables *A* for which $\tau_{A}\Delta_{H}\leq \hbar /2$. If instead we impose that the operator function ΔM(ρ)/τ is defined in such a way that $a_{\tau }\leq 1$, i.e.,

(26) $$\tau \geq \hbar \Delta_{M}/2k_{B}\Delta_{H}\;,$$

then we obtain that even in dissipative dynamics the usual time–energy uncertainty relations are never violated by observables *A* commuting with *H*. In Section 8 we will consider a numerical example for a case with non-constant τ given by Equation (26) with strict equality, for a qualitative comparison with the same case with constant τ.

However, in general, if the dynamics is dissipative [ΔM(ρ)/τ≠0] there are density operators for which $|c_{F\;H}+a_{\tau }\;r_{\;F\;M}|>1$ so that $\tau_{F}\Delta_{H}$ takes a value less than ℏ/2 and thus the usual time–energy uncertainty relation is violated. The sharpest general time–energy uncertainty relation that is always satisfied when both Hamiltonian and dissipative dynamics are active is (proof in Section 5)

(27) $$\frac{\hbar^{2}/4}{\tau_{F}^{2}\sigma_{\;H\;H}}\leq 1+a_{\tau }^{2}+2a_{\tau }c_{\;M\;H}\;,$$

which may also take the equivalent form

(28) $$\frac{\tau_{F}^{2}\sigma_{\;H\;H}}{\hbar^{2}/4}+\frac{\tau_{F}^{2}\sigma_{\;M\;M}}{k_{B}^{2}\tau^{2}\left(\rho \right)}+\frac{\tau_{F}^{2}\Delta_{M}\Delta_{H}c_{\;M\;H}}{k_{B}\tau \;\hbar /4}\geq 1\;.$$

The upper bound in the rate of change of property 〈F〉 becomes

(29) $$\Delta_{F}\sqrt{\frac{\sigma_{\;H\;H}}{\hbar^{2}/4}+\frac{\sigma_{\;M\;M}}{k_{B}^{2}\tau }+\frac{\Delta_{M}\Delta_{H}c_{\;M\;H}}{k_{B}\tau \;\hbar /4}}\;.$$

As anticipated, because the dissipative term in Equation (4) implies an additional dynamical mechanism, this bound (29), valid for the particular nonunitary dynamics we are considering, is higher than the standard bound valid in unitary hamiltonian dynamics, given by $2\Delta_{F}\Delta_{H}/\hbar$. For observables commuting with *H*, however, (25) provides the sharper general bound $\Delta_{F}\Delta_{M}/k_{B}\tau$, solely due to dissipative dynamics, which is lower than (29).

Because in general $|c_{\;M\;H}|<1$, (28) obviously implies the less precise relation

(30) $$\frac{\hbar^{2}/4}{\tau_{F}^{2}\sigma_{\;H\;H}}\leq {\left(1+a_{\tau }\right)}^{2}\;.$$

However, as for the dynamics we discuss in Section 7, if the Massieu operator ΔM(ρ) is a linear combination (with coefficients that may depend nonlinearly on ρ) of operators that commute with either ρ or *H*, then it is easy to show that $c_{\;M\;H}=0$. Therefore, in such important case, (28) becomes

(31) $$\frac{\hbar^{2}/4}{\tau_{F}^{2}\sigma_{\;H\;H}}\leq 1+a_{\tau }^{2}\;,$$

clearly sharper than (30). If in addition ΔM(ρ)/τ is such that (26) is satisfied, then (31) implies $\tau_{F}\Delta_{H}\geq \hbar /2\sqrt{2}$.

## 4. Characteristic Time of the Rate of Entropy Change

We now consider the entropy functional $\langle S\rangle =\mathrm{Tr}\left(\rho S\right)=-k_{B}\mathrm{Tr}\left(\rho \ln\rho \right)=-k_{B}\left(\sqrt{\rho }\left|\sqrt{\rho }\ln{\left(\sqrt{\rho }\right)}^{2}\right.\right)$ and its rate of change, which using Equations (4) and (8) may be written as

(32) $$d\mathrm{Tr}\left(\rho S\right)/dt=2\left(\left.\sqrt{\rho }S\right|\sqrt{\rho }E\left(\rho \right)\right)=\langle \Delta S\Delta M\rangle /k_{B}\tau =\Delta_{S}\Delta_{M}\;r_{\;S\;M}/k_{B}\tau \;,$$

where *S* is the entropy operator defined as follows

(33) $$S=-k_{B}P_{\mathrm{Ran}\rho }\ln\rho =-k_{B}\ln\left(\rho +P_{\mathrm{Ker}\rho }\right)\;,$$

where $P_{\mathrm{Ran}\rho }$ and $P_{\mathrm{Ker}\rho }$ are the projection operators onto the range and kernel of ρ. Operator *S*, introduced in [58,62], is always well defined for any $\rho \geq \rho^{2}$, even if some eigenvalues of ρ are zero. It is the null operator when $\rho^{2}=\rho$. In models where *S* is always multiplied by ρ or $\sqrt{\rho }$, the operators $P_{\mathrm{Ran}\rho }$ (or $P_{\mathrm{Ker}\rho }$) in Equation (33) could be omitted, because in general $\rho S=-k_{B}\rho \ln\rho$ and $\sqrt{\rho }S=-k_{B}\sqrt{\rho }\ln\rho$. But, in models of decoherence and composite systems based on the LSEA equation of motion proposed in [59], further discussed in [100], and applied for example in [68,69], their role is important because the LSEA master evolution equation involves the operators

(34) $$\begin{array}{c} {\left(H\right)}^{J}={\mathrm{Tr}}_{\bar{J}}\left[\left(I_{J}^{}⊗\rho_{\bar{J}}\right)H\right]\;, \end{array}$$

(35) $$\begin{array}{c} {\left(S\right)}^{J}={\mathrm{Tr}}_{\bar{J}}\left[\left(I_{J}^{}⊗\rho_{\bar{J}}\right)S\right]\;, \end{array}$$

that we call “locally perceived overall-system energy operator” and “locally perceived overall-system entropy operator,” respectively, associated with a mean-field-like measure of how the overall-system energy and entropy operators, *H* and *S*, are “perceived” locally within the *J*-th constituent subsystem. The symbol $\bar{J}$ denotes the composite of all subsystems except the J-th one. As discussed in full details in [59,100] the dissipative term in our LSEA master equation points in the direction of the local constrained gradient of the “locally perceived overall-system entropy” Tr_*J*_[*ρ*_*J*_(*S*)^*J*^], constrained by the condition of orthogonality with respect to the local gradient of the “locally perceived overall-system energy” Tr_*J*_[*ρ*_*J*_(*H*)^*J*^]. Operators (*S*)^*J*^ and, hence, the LSEA models just mentioned, would not be well defined without $P_{\mathrm{Ran}\rho }$ (or $P_{\mathrm{Ker}\rho }$) in Equation (33).

Interestingly, the rate of entropy change, being proportional to the correlation coefficient between entropy measurements and *M* measurements, under the assumptions made so far, may be positive or negative, depending on how ΔM(ρ) is defined, i.e., depending on the specifics of the physical model in which Equation (4) is adopted.

The characteristic time of change of the entropy functional, defined as

(36) $$\tau_{S}=\Delta_{S}/\left|d\langle S\rangle /dt\right|\;,$$

gives rise to the following equivalent exact time–energy uncertainty relations

(37) $$\frac{\hbar /2}{\tau_{S}\Delta_{H}}=a_{\tau }\;\left|r_{\;S\;M}\right|\leq a_{\tau }\;,$$

(38) $$\frac{k_{B}\tau }{\tau_{S}\Delta_{M}}=\left|r_{\;S\;M}\right|\leq 1\;,$$

where $r_{\;S\;M}$ is defined as in (16) using operators ΔM(ρ) and ΔS=S−〈S〉. The physical interpretation of (38) is that the entropy cannot change in time at a rate faster than $\Delta_{S}\Delta_{M}/k_{B}\tau$, as immediately obvious also from (32).

We notice from (37) that if the nonequilibrium Massieu operator satisfies condition (26) then $a_{\tau }\leq 1$ and, therefore, the characteristic time of entropy change, $\tau_{S}$, satisfies the usual uncertainty relation $\tau_{S}\Delta_{H}\geq \hbar /2$ and the rate of entropy change cannot exceed $2\Delta_{S}\Delta_{H}/\hbar$.

We conclude this Section by noting that, in general, the equality in (37) may be used to rewrite Relation (27) in the form

(39) $$\frac{a_{\tau }}{1+a_{\tau }}\left|r_{\;S\;M}\right|\tau_{S}\leq \tau_{F}\frac{\sqrt{1+a_{\tau }^{2}+2a_{\tau }c_{\;M\;H}}}{1+a_{\tau }}\leq \tau_{F}\;,$$

where the last inequality follows from $|c_{\;M\;H}|\leq 1$. This relation shows, on one hand, that the entropy change characteristic time $\tau_{S}$ is not necessarily the shortest among the characteristic times $\tau_{F}$ associated with observables of the type 〈F〉=Tr(ρF) according to the Mandelstam–Tamm definition (19). On the other hand, it also shows that the left-hand side defines a characteristic-time functional

(40) $$\tau_{U\;D}=\frac{a_{\tau }}{1+a_{\tau }}\left|r_{\;S\;M}\right|\tau_{S}\leq \tau_{F}\;,$$

which constitutes a general lower bound for all $\tau_{F}$’s, and may therefore be considered the shortest characteristic time of simultaneous unitary+dissipative dynamics as described by Equation (4). This observation prompts the discussion in the next section.

## 5. Shortest Characteristic Times for Purely-Unitary and Purely-Dissipative Dynamics

The Mandelstam–Tamm definition (19) of characteristic times has been criticized for various reasons (see for example References [101,102,103]) mainly related to the fact that depending on which observable *F* is investigated, as seen by inspecting (23), the bound $\tau_{F}\geq \hbar /2\Delta_{H}$ may be very poor whenever $c_{\;F\;H}^{2}$ is much smaller than 1.

Therefore, different attempts have been made to define characteristic times that (1) refer to the quantum system as a whole rather than to some particular observable, and (2) bound all the particular $\tau_{F}$’s from below. Notable examples are the characteristic times $\tau_{ES}$ and $\tau_{LK}$, respectively defined by Eberly and Singh [101] and Leubner and Kiener [102].

Here, however, we consider the shortest characteristic times that emerge from the following geometrical observations. The functional $\Delta_{F}$ may be interpreted as the norm of $\sqrt{\rho }\Delta F$ (viewed as a vector in L(H)) in the sense that it equals $\sqrt{\left(\sqrt{\rho }\Delta F|\sqrt{\rho }\Delta F\right)}$, therefore, we may use it to define the (generally non hermitian) unit norm vector in L(H)

(41) $${\tilde{F}}_{\rho }=\sqrt{\rho }\Delta F/\Delta_{F}\;.$$

As a result, Equation (13) may be rewritten in the form

(42) $$\frac{1}{\Delta_{F}}\frac{d\langle F\rangle }{dt}=\frac{\Delta_{H}}{\hbar /2}\;\left({\tilde{F}}_{\rho }|i{\tilde{H}}_{\rho }\right)+\frac{\Delta_{M}}{k_{B}\tau }\;\left({\tilde{F}}_{\rho }|{\tilde{M}}_{\rho }\right)=\left({\tilde{F}}_{\rho }|C\right)\;,$$

where for shorthand we define the operator

(43) $$C=i\frac{\Delta_{H}{\tilde{H}}_{\rho }}{\hbar /2}+\frac{\Delta_{M}{\tilde{M}}_{\rho }}{k_{B}\tau }=2\sqrt{\rho }E\left(\rho \right)\;,$$

directly related [see Equation (7)] with the evolution operator function E(ρ) defined in Section 2, which determines the rates of change of all linear functionals of the state operator ρ, i.e., all observables of the linear type Tr(ρF), by its projection onto the respective directions ${\tilde{F}}_{\rho }$.

Each characteristic time $\tau_{F}$ can now be written as

(44) $$\tau_{F}=\Delta_{F}/|d\langle F\rangle /dt|=1/|\left({\tilde{F}}_{\rho }|C\right)|\;.$$

Because ${\tilde{F}}_{\rho }$ is unit norm, $\left|({\tilde{F}}_{\rho }|C)\right|$ is bounded by the value attained for an operator ${\tilde{F}}_{\rho }$ that has the same ‘direction’ in L(H) as operator *C*, i.e., for

(45) $${\tilde{F}}_{\rho }=\pm C/\sqrt{\left(C|C\right)}\;,$$

in which case $|\left({\tilde{F}}_{\rho }|C\right)|=\sqrt{\left(C|C\right)}=\sqrt{\mathrm{Tr}(C^{†}C)}$. Thus we conclude that, for any, *F*,

(46) $$1/\sqrt{\left(C|C\right)}\leq \tau_{F}\;,$$

and, therefore, we introduce the shortest characteristic time for the combined unitary+dissipative dynamics described by Equation (4),

(47) $$\tau_{U\;D}=1/\sqrt{\left(C|C\right)}\;,$$

which binds from below all $\tau_{F}$’s. From (43) and (46), and the identities $\left(i{\tilde{H}}_{\rho }|i{\tilde{H}}_{\rho }\right)=\left({\tilde{M}}_{\rho }|{\tilde{M}}_{\rho }\right)=1$ and $\left(i{\tilde{H}}_{\rho }|{\tilde{M}}_{\rho }\right)=\left({\tilde{M}}_{\rho }|i{\tilde{H}}_{\rho }\right)=c_{\;M\;H}$ we obtain

(48) $$\begin{array}{ccc} \frac{1}{\tau_{F}^{2}}\leq \frac{1}{\tau_{U\;D}^{2}} & = & \left(C|C\right)=\frac{\sigma_{\;H\;H}}{\hbar^{2}/4}+\frac{\sigma_{\;M\;M}}{k_{B}^{2}\tau^{2}\left(\rho \right)}+\frac{\Delta_{M}\Delta_{H}c_{\;M\;H}}{k_{B}\tau \;\hbar /4}\\ & = & \frac{\sigma_{\;H\;H}}{\hbar^{2}/4}\left(1+a_{\tau }^{2}+2\;a_{\tau }c_{\;M\;H}\right)\;, \end{array}$$

which proves relations (27) and (28).

For nondissipative (purely Hamiltonian, unitary) dynamics the same reasoning (or substitution of τ=∞, $a_{\tau }=0$ in the above relations) leads to the definition of the shortest characteristic time of unitary dynamics

(49) $$\tau_{U}=\hbar /2\Delta_{H}\;,$$

with which the usual time–energy relation reduces to

(50) $$\tau_{F}\geq \tau_{U}\;.$$

Its physical meaning is that when the energy dispersion (or uncertainty or spread) $\Delta_{H}$ is small, $\tau_{U}$ is large and $\tau_{F}$ must be larger for all observables *F*, therefore, the mean values of all properties change slowly [15,104,105], i.e., the state ρ has a long lifetime. In other words, states with a small energy spread cannot change rapidly with time. Conversely, states that change rapidly due to unitary dynamics, necessarily have a large energy spread.

Another interesting extreme case obtains from Equation (4) when ΔM(ρ) is such that the condition [ρ,H]=0 implies [ΔM(ρ),H]=0 for any ρ, as for the steepest-entropy-ascent dynamics discussed in Section 7 and Section 8. In this case, it is easy to see that if the state operator ρ commutes with *H* at one instant of time then it commutes with *H* at all times and, therefore, the entire time evolution is purely dissipative. Then, the reasoning above leads to the definition of the shortest characteristic time of purely dissipative evolution

(51) $$\tau_{D}=k_{B}\tau /\Delta_{M}\;.$$

It is noteworthy that $\tau_{D}$ can be viewed as the characteristic time associated not with the (generally nonlinear) Massieu functional 〈M〉=Tr(ρM(ρ)) but with the linear functional 〈A〉=Tr(ρA) corresponding to the time-independent operator *A* which at time *t* happens to coincide with M(ρ(t)).

For purely dissipative dynamics, the bound $\tau_{F}\geq \tau_{D}=k_{B}\tau /\Delta_{M}$ implies that when $\Delta_{M}/k_{B}\tau$, i.e., the ratio between the uncertainty in our generalized nonequilibrium Massieu observable represented by operator *M* and the intrinsic dissipation time τ, is small, then $\tau_{D}$ is large and $\tau_{F}$ must be larger for all observables *F*, therefore, the state ρ has a long lifetime. This may be a desirable feature in quantum computing applications where the interest is in engineering states ρ that preserve the entanglement of component subsystems. Conversely, if some observable changes rapidly, $\tau_{F}$ is small and since $\tau_{D}$ must be smaller, we conclude that the spread $\Delta_{M}$ (more precisely, the ratio $\Delta_{M}/k_{B}\tau$) must be large.

In terms of $\tau_{U}$ and $\tau_{D}$ we can rewrite (20), (38) and (48) as

(52) $$a_{\tau }=\tau_{U}/\tau_{D}\;,$$

(53) $$\frac{1}{\tau_{S}}=\frac{|r_{\;S\;M}|}{\tau_{D}}\leq \frac{1}{\tau_{D}}\;,$$

(54) $$\begin{array}{ccc} \frac{1}{\tau_{F}^{2}} & = & {\left(\frac{c_{F\;H}}{\tau_{U}}+\frac{r_{\;F\;M}}{\tau_{D}}\right)}^{2}\\ & \leq & \frac{1}{\tau_{U\;D}^{2}}=\frac{1}{\tau_{U}^{2}}+\frac{1}{\tau_{D}^{2}}+\frac{2\;c_{\;M\;H}}{\tau_{U}\tau_{D}}\\ & \leq & {\left(\frac{1}{\tau_{U}}+\frac{1}{\tau_{D}}\right)}^{2}. \end{array}$$

Equation (53) implies that the entropy cannot change rapidly with time if the ratio $\Delta_{M}\left(\rho \right)/k_{B}\tau$ is not large. The first equality in (54) follows from $\left({\tilde{F}}_{\rho }|i{\tilde{H}}_{\rho }\right)=c_{F\;H}$ and $\left({\tilde{F}}_{\rho }|{\tilde{M}}_{\rho }\right)=r_{\;F\;M}$, which also imply that Equation (42) may take the form

(55) $$\frac{d\langle F\rangle }{dt}=\Delta_{F}\left(\frac{c_{F\;H}}{\tau_{U}}+\frac{r_{\;F\;M}}{\tau_{D}}\right)\;,$$

and operator *C* defined in (43) takes also the forms

(56) $$C=i\frac{{\tilde{H}}_{\rho }}{\tau_{U}}+\frac{{\tilde{M}}_{\rho }}{\tau_{D}}=i\frac{\sqrt{\rho }\Delta H}{\Delta_{H}\tau_{U}}+\frac{\sqrt{\rho }\Delta M}{\Delta_{M}\tau_{D}}\;,$$

and its norm is $\sqrt{1/\tau_{U}^{2}+1/\tau_{D}^{2}+2c_{\;M\;H}/\tau_{U}\tau_{D}}$.

Similarly, the rate of entropy change (32) takes the form

(57) $$\frac{d\langle S\rangle }{dt}=\frac{\Delta_{S}}{\tau_{D}}\;\left({\tilde{S}}_{\rho }\;|\;{\tilde{M}}_{\rho }\right)=\frac{\Delta_{S}\;r_{\;S\;M}}{\tau_{D}}$$

which, because $|r_{\;S\;M}|\leq 1$, implies the bounds [equivalent to (38) and (53)],

(58) $$-\frac{\Delta_{S}}{\tau_{D}}\leq \frac{d\langle S\rangle }{dt}\leq \frac{\Delta_{S}}{\tau_{D}}\;.$$

## 6. Occupation Probabilities

An important class of observables for a quantum system are those associated with the projection operators. For example, for pure states evolving unitarily, the mean value 〈P〉=Tr(ρ(t)P) where $P=|\phi_{0}\rangle \langle \phi_{0}|=\rho \left(0\right)$ represents the survival probability of the initial state, and is related to several notions of lifetimes [15,104,105].

We do not restrict our attention to pure states, and we discuss first results that hold for any projector *P* associated with a yes/no type of measurement. Let $P=P^{†}=P^{2}$ be an orthogonal projector onto the *g*-dimensional subspace PH of H. Clearly, g=Tr(P), the variance 〈ΔPΔP〉=p(1−p) where p=〈P〉=Tr(ρP) denotes the mean value and represents the probability in state ρ of obtaining a ‘yes’ result upon measuring the associated observable. The characteristic time of the rate of change of this occupation probability is defined according to (19) by

(59) $$\begin{array}{ccc} \frac{1}{\tau_{P}} & = & \frac{\left|dp/dt\right|}{\sqrt{p\;(1-p)}}=2\left|\frac{d}{dt}\arccos\left(\sqrt{p}\right)\right|\\ & = & 2\left|\frac{d}{dt}\arcsin\left(\sqrt{p}\right)\right|\leq \frac{1}{\tau_{U\;D}}\;, \end{array}$$

where the inequality follows from (48). Therefore,

(60) $$-\frac{1}{2\tau_{U\;D}}\leq \frac{d}{dt}\arccos\left(\sqrt{p}\right)\leq \frac{1}{2\tau_{U\;D}}\;,$$

or, over any finite time interval of any time history p(t),

(61) $$\left|\arccos\left(\sqrt{p(t_{2})}\right)-\arccos\left(\sqrt{p(t_{1})}\right)\right|\leq \left|\int_{t_{1}}^{t_{2}}\frac{dt^{\prime }}{2\tau_{U\;D}\left(t^{\prime }\right)}\right|\;.$$

This result generalizes the results on lifetimes obtained in [103] where the focus is restricted to full quantum decay [p(∞)≈0] of an initially fully populated state [p(0)≈1] and $\tau_{U}$ (here $\tau_{U\;D}$) is assumed constant during the time interval. It is also directly related to some of the results in [15,104,105], where a number of additional inequalities and bounds on lifetimes are obtained for unitary dynamics, and may be straightforwardly generalized to the class of simultaneous unitary/dissipative dynamics described by our Equation (4).

Because p(1−p) attains its maximum value when p=1/2, we also have the inequality

(62) $$\left|\frac{dp}{dt}\right|\leq \frac{1}{2\tau_{U\;D}}\;.$$

which, analogously to what noted in [103], implies that no full decay nor full population can occur within a time $2\tau_{U\;D}$, so that this time may be interpreted as a limit to the degree of instability of a quantum state.

Next, we focus on the projectors onto the eigenspaces of the Hamiltonian operator *H*, assumed time-independent. Let us write its spectral expansion as $H=\sum_{n}e_{n}P_{\;e_{\;n}}$ where $e_{n}$ is the *n*-th eigenvalue and $P_{\;e_{\;n}}$ the projector onto the corresponding eigenspace. Clearly, $HP_{\;e_{\;n}}=e_{n}P_{\;e_{\;n}}$, $P_{\;e_{\;n}}P_{\;e_{\;m}}=\delta_{nm}P_{\;e_{\;n}}$, $g_{n}=\mathrm{Tr}\left(P_{\;e_{\;n}}\right)$ is the degeneracy of eigenvalue $e_{n}$, $p_{n}=\langle P_{\;e_{\;n}}\rangle =\mathrm{Tr}\left(\rho P_{\;e_{\;n}}\right)$ the occupation probability of energy level $e_{n}$, $\langle \Delta P_{\;e_{\;n}}\Delta P_{\;e_{\;m}}\rangle =p_{n}\;\left(\delta_{nm}-p_{m}\right)$ the covariance of pairs of occupations, and $\langle \Delta P_{\;e_{\;n}}\Delta P_{\;e_{\;n}}\rangle =p_{n}\;\left(1-p_{n}\right)$ the variance or fluctuation of the *n*-th occupation. Because $[P_{\;e_{\;n}},H]=0$, $c_{P_{\;e_{\;n}}\;\;H}=0$ and by (55) we have

(63) $$\frac{dp_{n}}{dt}=\Delta_{P_{\;e_{\;n}}}\frac{r_{P_{\;e_{\;n}}\;\;M}}{\tau_{D}}\;,$$

and the corresponding characteristic time is

(64) $$\frac{1}{\tau_{P_{\;e_{\;n}}}}=\frac{|r_{P_{\;e_{\;n}}\;\;M}|}{\tau_{D}}\leq \frac{1}{\tau_{D}}\;.$$

Energy level occupation probabilities $p_{n}$ are used in Section 8 for numerical illustration/validation of inequalities (64) within the steepest-entropy-ascent dynamical model outlined in the next Section.

## 7. Example. Steepest-Entropy-Ascent Master Equation for Conservative Dissipative Dynamics

So far we have not assumed an explicit form of the operator ΔM(ρ) except for the condition that it maintains ρ unit trace ((3) or (8)) and nonnnegative definite. In this section, we illustrate the above results by further assuming a particular form of steepest-entropy-ascent, conservative dissipative dynamics. For our generalized nonequilibrium Massieu operator we assume the expression

(65) $$\Delta M\left(\rho \right)=\Delta S-\Delta H^{\prime }\left(\rho \right)/\theta \left(\rho \right)\;,$$

where *S* is the entropy operator defined in Equation (33),

(66) $$\Delta H^{\prime }\left(\rho \right)=\Delta H-\nu \left(\rho \right)\cdot \Delta N\;,$$

*H* is the Hamiltonian operator, $N=\{N_{1},\cdots ,N_{r}\}$ a (possibly empty) set of operators commuting with *H* that we call non-Hamiltonian generators of the motion (for example, the number-of-particles operators or a subset of them, or the momentum component operators for a free particle) and that must be such that operators $\sqrt{\rho }\Delta H$ and $\sqrt{\rho }\Delta N$ are linearly independent, and—most importantly—θ(ρ) and $\nu \left(\rho \right)=\{\nu_{1}\left(\rho \right),\cdots ,\nu_{r}\left(\rho \right)\}$ are a set of real functionals defined for each ρ by the solution of the following system of linear equations

(67) $$\begin{array}{ccc} \langle \Delta S\Delta H\rangle \;\theta +\sum_{i=1}^{r}\langle \Delta N_{i}\Delta H\rangle \;\nu_{i} & = & \langle \Delta H\Delta H\rangle \;, \end{array}$$

(68) $$\begin{array}{ccc} \langle \Delta S\Delta N_{j}\rangle \;\theta +\sum_{i=1}^{r}\langle \Delta N_{i}\Delta N_{j}\rangle \;\nu_{i} & = & \langle \Delta H\Delta N_{j}\rangle \;, \end{array}$$

which warrant the conditions that 〈ΔHΔM〉=0 and $\langle \Delta N_{j}\Delta M\rangle =0$, and hence that the mean values Tr(ρH) and Tr(ρN) are maintained time invariant by the dissipative term of the resulting SEA master equation [Equation (4) together with Equations (65)–(68)].

As a result, our assumption may be rewritten as follows

(69) ΔM(ρ)=M(ρ)−ITr[ρM(ρ)]

where *I* is the identity and the nonequilibrium Massieu operator M(ρ) is the following nonlinear function of ρ

(70) $$M\left(\rho \right)=S\left(\rho \right)-\frac{H}{\theta (\rho )}+\frac{\nu (\rho )\cdot N}{\theta (\rho )}\;,$$

and we note that at a thermodynamic equilibrium (Gibbs) state,

(71) $$\rho_{e}=\frac{1}{Z}\exp\left(-\frac{H-\mu_{e}\cdot N}{T_{e}}\right)\;,$$

its mean value belongs to the family of entropic characteristic functions introduced by Massieu [106], i.e.,

(72) $${\langle M\rangle }_{e}={\langle S\rangle }_{e}-\frac{{\langle H\rangle }_{e}}{T_{e}}+\frac{\mu_{e}\cdot {\langle N\rangle }_{e}}{T_{e}}\;,$$

where ${\langle S\rangle }_{e}$, ${\langle H\rangle }_{e}$, ${\langle N\rangle }_{e}$, $T_{e}=\theta \left(\rho_{e}\right)$ and $\mu_{e}=\nu \left(\rho_{e}\right)$ are the (grand canonical) equilibrium entropy, energy, amounts of constituents, temperature and chemical potentials, respectively.

Notice that operator *M*, its eigenvalues and its mean value Tr(ρM) for a given state ρ, that we first termed “nonequilibrium Massieu operator” in References [62,94,107], differ substantially from the “nonequilibrium Massieu potentials” defined recently in References [108,109]. Their nonequilibrium Massieu construct is defined by the difference between the entropy and a linear combination of the conserved properties, with coefficients that are weighted averages of the fixed temperatures and other entropic potentials of the reservoirs interacting with the system. In our nonequilibrium Massieu construct, instead, the coefficients θ and ν of the linear combination are truly nonequilibrium functionals of the state ρ, that evolve in time with ρ, and that only when the system has relaxed to equilibrium can be identified with the inverse temperature 1/T of the system and the entropic potentials −μ/T of the other conserved properties.

The non-Hamiltonian generators of the motion represent the other conserved properties of the system, however, this condition may be relaxed in the framework of a resource theory of a quantum thermodynamic subsystem that, via the Hamiltonian part of the master equation, exchanges with other systems or a thermal bath some non-commuting quantities or “charges”, as recently envisioned in Reference [110].

Operators $\sqrt{\rho }\Delta H^{\prime }$ and $\sqrt{\rho }\Delta M$ are always orthogonal to each other, in the sense that $\langle \Delta M\Delta H^{\prime }\rangle =0$ for every ρ. It follows that, in general, $\langle \Delta S\Delta H^{\prime }\rangle =\langle \Delta H^{\prime }\Delta H^{\prime }\rangle /\theta$,

(73) $$\langle \Delta S\Delta M\rangle =\langle \Delta M\Delta M\rangle =\langle \Delta S\Delta S\rangle -\frac{\langle \Delta H^{\prime }\Delta H^{\prime }\rangle }{\theta^{2}\left(\rho \right)}\geq 0\;,$$

and hence the rate of entropy generation (32) is always strictly positive except for 〈ΔMΔM〉=0 (which occurs iff $\sqrt{\rho }\Delta M=0$), i.e., for $\sqrt{\rho_{\mathrm{nd}}}\Delta S_{\mathrm{nd}}=\left(\sqrt{\rho_{\mathrm{nd}}}\Delta H-\mu_{\mathrm{nd}}\cdot \sqrt{\rho_{\mathrm{nd}}}\Delta N\right)/T_{\mathrm{nd}}$, for some real scalars $T_{\mathrm{nd}}$ and $\mu_{\mathrm{nd}}$, that is, for density operators (that we call non-dissipative [58,62,94,107]) of the following Gibbs (or partially Gibbs, if B≠I) form

(74) $$\rho_{\mathrm{nd}}=\frac{B\exp[-\left(H-\mu_{\mathrm{nd}}\cdot N\right)/k_{B}T_{\mathrm{nd}}]B}{\mathrm{Tr}B\exp[-\left(H-\mu_{\mathrm{nd}}\cdot N\right)/k_{B}T_{\mathrm{nd}}]}\;,$$

where *B* is any projection operator on H ($B^{2}=B$).

The nonlinear functional

(75) $$\theta \left(\rho \right)=\frac{\sigma_{\;H^{\prime }\;H^{\prime }}}{\sigma_{\;S\;H^{\prime }}}=\frac{\Delta_{H^{\prime }}}{\Delta_{S}\;r_{\;S\;H^{\prime }}}$$

may be interpreted in this framework as a natural generalization to nonequilibrium of the temperature, at least insofar as for t→+∞, while the state operator ρ(t) approaches a non-dissipative operator of form (74), θ(ρ(t)) approaches smoothly the temperature $T_{\mathrm{nd}}$ of the non-dissipative thermodynamic equilibrium (stable, if B=I, or unstable, if B≠I) or of the unstable limit cycle (if [B,H]≠0), and −ν(ρ(t))/θ(ρ(t)) approach smoothly the corresponding entropic potentials $-\mu_{\mathrm{nd}}/T_{\mathrm{nd}}$.

Because here we assumed that *H* always commutes with *M*, $c_{\;M\;H}=0$ and $(\tilde{M}|i\tilde{H})=0$, which means that $\sqrt{\rho }\Delta M\left(\rho \right)$ is always orthogonal to $i\sqrt{\rho }\Delta H$. This reflects the fact that the direction of steepest-entropy-ascent is orthogonal to the (constant entropy) orbits that characterize purely Hamiltonian (unitary) motion (which maintains the entropy constant by keeping invariant each eigenvalue of ρ).

Here, for simplicity, we have assumed that dissipation pulls the state in the direction of steepest-entropy-ascent with respect to the uniform Fisher–Rao metric (see [62]). However, we have discussed elsewhere (see [34,35]) that, in general, a most important and characterizing feature of the nonequilibrium states of a system is the metric with respect to which the system identifies the direction of steepest-entropy-ascent. In most cases, it is a non-uniform metric, such as for a material with a nonisotropic thermal conductivity or, in the quantum framework, for a spin system in a magnetic field that near equilibrium obeys the Bloch equations [111] with different relaxation times along the field and normal to the field.

Inequality (73), which follows from $r_{\;S\;M}^{2}\leq 1$, implies that $\sigma_{\;M\;M}\leq \sigma_{\;S\;S}$ and $0\leq r_{\;S\;M}=\Delta_{M}/\Delta_{S}\leq 1$ or, equivalently,

(76) $$\tau_{K}=k_{B}\tau /\Delta_{S}\leq \tau_{D}\;,$$

where for convenience we define the characteristic time $\tau_{K}$, which is simply related to the entropy uncertainty, but cannot be attained by any rate of change, being shorter than $\tau_{D}$. In addition, we have the identities

(77) $$r_{\;S\;M}^{2}=\frac{\sigma_{\;M\;M}}{\sigma_{\;S\;S}}=\frac{\tau_{K}^{2}}{\tau_{D}^{2}}=\frac{\tau_{K}}{\tau_{S}}=1-\frac{\sigma_{\;H^{\prime }\;H^{\prime }}}{\theta^{2}\sigma_{\;S\;S}}=1-r_{\;S\;H^{\prime }}^{2}\;,$$

and, from $r_{\;S\;H^{\prime }}^{2}\leq 1$, the bounds

(78) $$\left|\theta \right|\geq \frac{\Delta_{H^{\prime }}}{\Delta_{S}}\;\mathrm{or}\;-\frac{\Delta_{S}}{\Delta_{H^{\prime }}}\leq \frac{1}{\theta }\leq \frac{\Delta_{S}}{\Delta_{H^{\prime }}}\;,$$

where the equality $\left|\theta \right|=\Delta_{H^{\prime }}/\Delta_{S}$ holds when and only when the state is non-dissipative [Equation (74)]. Additional bounds on our generalized nonequilibrium temperature θ obtain by combining (77) with the inequality $4r_{\;S\;M}^{2}\left(1-r_{\;S\;M}^{2}\right)\leq 1$ (which clearly holds because $r_{\;S\;M}^{2}\leq 1$), to obtain $4r_{\;S\;M}^{2}r_{\;S\;H^{\prime }}^{2}\leq 1$ and, therefore,

(79) $$\frac{2\Delta_{M}\Delta_{H^{\prime }}}{\left|\theta \right|\sigma_{\;S\;S}}\leq 1\;\mathrm{or}\;-\frac{\sigma_{\;S\;S}}{2\Delta_{M}\Delta_{H^{\prime }}}\leq \frac{1}{\theta }\leq \frac{\sigma_{\;S\;S}}{2\Delta_{M}\Delta_{H^{\prime }}}\;.$$

At equilibrium, $\Delta_{M}=0$ and (79) implies no actual bound on θ, but in nonequilibrium states bounds (79) may be tighter than (78), as illustrated by the numerical example in Section 8.

Notice that whereas in steepest-entropy-ascent dynamics $\tau_{K}$ is always shorter than $\tau_{D}$ and obeys the identity

(80) $$\tau_{S}\tau_{K}=\tau_{D}^{2}\;,$$

in general it is not necessarily shorter than $\tau_{D}$ and obeys the identity

(81) $$\frac{\Delta_{M}}{\Delta_{S}}\frac{\tau_{D}^{2}}{\tau_{S}\tau_{K}}=\left|r_{\;S\;M}\right|\;.$$

In summary, we conclude that within steepest-entropy-ascent, conservative dissipative quantum dynamics, the general uncertainty relations (28), (37) and (38) that constitute the main results of this paper, yield the time–energy/time-Massieu uncertainty relation

(82) $${\left(\frac{\tau_{F}\Delta_{H}}{\hbar /2}\right)}^{2}+{\left(\frac{\tau_{F}\Delta_{M}}{k_{B}\tau }\right)}^{2}\geq 1\;\mathrm{or}\;\frac{\tau_{F}^{2}}{\tau_{U}^{2}}+\frac{\tau_{F}^{2}}{\tau_{D}^{2}}\geq 1\;,$$

which implies the interesting time–energy and time–entropy uncertainty relation

(83) $${\left(\frac{\tau_{F}\Delta_{H}}{\hbar /2}\right)}^{2}+{\left(\frac{\tau_{F}\Delta_{S}}{k_{B}\tau }\right)}^{2}\geq 1\;\mathrm{or}\;\frac{\tau_{F}^{2}}{\tau_{U}^{2}}+\frac{\tau_{F}^{2}}{\tau_{K}^{2}}\geq 1\;,$$

and the time–entropy uncertainty relation

(84) $$\frac{\tau_{K}}{\tau_{S}}=\frac{k_{B}\tau }{\tau_{S}\Delta_{S}}=r_{\;S\;M}^{2}\leq r_{\;S\;M}\leq 1\;,$$

which implies that the rate of entropy generation never exceeds $\sigma_{\;S\;S}/k_{B}\tau$, i.e.,

(85) $$\frac{d\langle S\rangle }{dt}=-k_{B}\frac{d}{dt}\mathrm{Tr}\left(\rho \ln\rho \right)=\frac{\sigma_{\;M\;M}}{k_{B}\tau }\leq \frac{\Delta_{S}\Delta_{M}}{k_{B}\tau }\leq \frac{\sigma_{\;S\;S}}{k_{B}\tau }\;.$$

If in addition the dynamics is purely dissipative, such as along a trajectory ρ(t) that commutes with *H* for every *t*, then (83) may be replaced by the time–entropy uncertainty relation

(86) $$\frac{\tau_{K}}{\tau_{F}}=\frac{k_{B}\tau }{\tau_{F}\Delta_{S}}\leq 1\;.$$

As shown in References [58,62], the dissipative dynamics generated by Equation (4) with ΔM(ρ) as just defined and a time-independent Hamiltonian *H*: (i) maintains $\rho \left(t\right)\geq \rho^{2}\left(t\right)$ at all times, both forward and backward in time for any initial density operator ρ(0) (see also [90,91]); (ii) maintains the cardinality of ρ(t) invariant; (iii) entails that the entropy functional is an *S*-function in the sense defined in [112] and therefore that maximal entropy density operators (Gibbs states) obtained from (74) with B=I are the only equilibrium states of the dynamics that are stable with respect to perturbations that do not alter the mean values of the energy and the other time invariants (if any): this theorem of the dynamics coincides with the Hatsopoulos-Keenan statement of the second law of thermodynamics [86]; (iv) entails Onsager reciprocity in the sense defined in [113]; (v) can be derived from a variational principle [90,91], equivalent to our steepest-entropy-ascent geometrical construction, by maximizing the entropy generation rate subject to the Tr(ρ), Tr(ρH), and Tr(ρN) conservation constraints and the additional constraint $\left(\sqrt{\rho }E|\sqrt{\rho }E\right)=c\left(\rho \right)$.

Operator $\sqrt{\rho }E$ is a ‘vector’ in L(H) and determines through its scalar product with $\sqrt{\rho }F$ and $\sqrt{\rho }S$ [Equations (7) and (32)] the rates of change of Tr(ρF) and Tr(ρS), respectively. From (32) and the Schwarz inequality ${\left(\sqrt{\rho }S|\sqrt{\rho }E\right)}^{2}\leq \left(\sqrt{\rho }S|\sqrt{\rho }S\right)\left(\sqrt{\rho }E|\sqrt{\rho }E\right)$, we see that for a given ρ, among all vectors $\sqrt{\rho }E$ with given norm $\left(\sqrt{\rho }E|\sqrt{\rho }E\right)=c\left(\rho \right)$, the one maximizing $\left(\sqrt{\rho }S|\sqrt{\rho }E\right)$ has the same direction as $\sqrt{\rho }S$. In general, along such direction Tr(ρH) and Tr(ρN) are not conserved because $\sqrt{\rho }S$ is not always orthogonal to $\sqrt{\rho }H$ and $\sqrt{\rho }N$. Instead, dynamics along the direction of steepest-entropy-ascent compatible with such conservation requirements, as first postulated and formulated in [57,58,62], obtains when $\sqrt{\rho }E$ has the direction of the component of $\sqrt{\rho }S$ orthogonal to $\sqrt{\rho }H$ and $\sqrt{\rho }N$. This is precisely how ΔM(ρ) is defined through Equations (65)–(68). See also Reference [100].

We finally note that assuming in Equation (4) a ΔM(ρ)/τ that satisfied Equation (26) with strict equality, we obtain the most dissipative (maximal entropy generation rate) dynamics in which the entropic characteristic time $\tau_{S}$ (Equation (36)) is always compatible with the time–energy uncertainty relation $\tau_{S}\Delta_{H}\geq \hbar /2$ and the rate of entropy generation is always given by $2\Delta_{M}\Delta_{H}/\hbar$.

The physical meaning of relations (28), (37), (38), (83), (84) are worth further investigations and experimental validation in specific contexts in which the dissipative behavior is correctly modeled by a dynamical law of form (4), possibly with ΔM(ρ)/τ of form (65). One such context may be the currently debated so-called “fluctuation theorems” [114,115,116,117] whereby fluctuations and, hence, uncertainties are measured on a microscopic system (optically trapped colloidal particle [118,119], electrical resistor [120]) driven at steady state (off thermodynamic equilibrium) by means of a work interaction, while a heat interaction (with a bath) removes the entropy being generated by irreversibility. Another such context may be that of pion-nucleus scattering, where available experimental data have recently allowed partial validation [121] of “entropic” uncertainty relations [122,123,124]. Yet another is within the model we propose in Reference [94] for the description of the irreversible time evolution of a perturbed, isolated, physical system during relaxation toward thermodynamic equilibrium by spontaneous internal rearrangement of the occupation probabilities. We pursue this example in the next section.

## 8. Numerical Results for Relaxation within a Single *N*-Level Qudit or a One-Particle Model of a Dilute Boltzmann Gas of *N*-Level Particles

To illustrate the time dependence of the uncertainty relations derived in this paper, we consider an isolated, closed system composed of noninteracting identical particles with single-particle eigenstates with energies $e_{i}$ for i=1, 2,..., *N*, where *N* is assumed finite for simplicity and the $e_{i}$’s are repeated in case of degeneracy, and we restrict our attention to the class of dilute-Boltzmann-gas states in which the particles are independently distributed among the *N* (possibly degenerate) one-particle energy eigenstates. This model is introduced in Reference [94], where we assume an equation of form (4) with ΔM(ρ) given by (65) with the further simplification that $\Delta H^{\prime }\left(\rho \right)=\Delta H$ so that our generalized nonequilibrium Massieu operator is simply

(87) M(ρ)=S−H/θ(ρ),

and, therefore,

(88) ΔM(ρ)=ΔS−ΔH/θ(ρ).

For simplicity and illustrative purposes, we focus on purely dissipative dynamics by considering a particular trajectory ρ(t) that commutes with *H* at all times *t*, assuming that *H* is time independent and has a nondegenerate spectrum. As a result, the energy-level occupation probabilities $p_{n}$ coincide with the eigenvalues of ρ, and the dynamical equation reduces to the simple form [94]

(89) $$\frac{dp_{n}}{dt}=-\frac{1}{\tau }\left[p_{n}\ln p_{n}+p_{n}\frac{\langle S\rangle }{k_{B}}+p_{n}\frac{e_{n}-\langle H\rangle }{k_{B}\theta }\right]\;,$$

where

(90) $$\begin{array}{ccc} \langle S\rangle & = & -k_{B}\sum_{n}p_{n}\ln p_{n}\;, \end{array}$$

(91) $$\begin{array}{ccc} \langle H\rangle & = & \sum_{n}p_{n}e_{n}\;, \end{array}$$

(92) $$\begin{array}{ccc} \theta & = & \sigma_{\;H\;H}/\sigma_{\;H\;S}\;, \end{array}$$

(93) $$\begin{array}{ccc} \sigma_{\;H\;H} & = & \sum_{n}p_{n}e_{n}^{2}-{\langle H\rangle }^{2}\;, \end{array}$$

(94) $$\begin{array}{ccc} \sigma_{\;H\;S} & = & -k_{B}\sum_{n}p_{n}e_{n}\ln p_{n}-\langle H\rangle \langle S\rangle \;. \end{array}$$

The same model describes relaxation to the Gibbs state of an *N*-level qudit with time independent Hamiltonian *H* from arbitrary initial states ρ(0) that commute with *H*.

**Figure 1:**
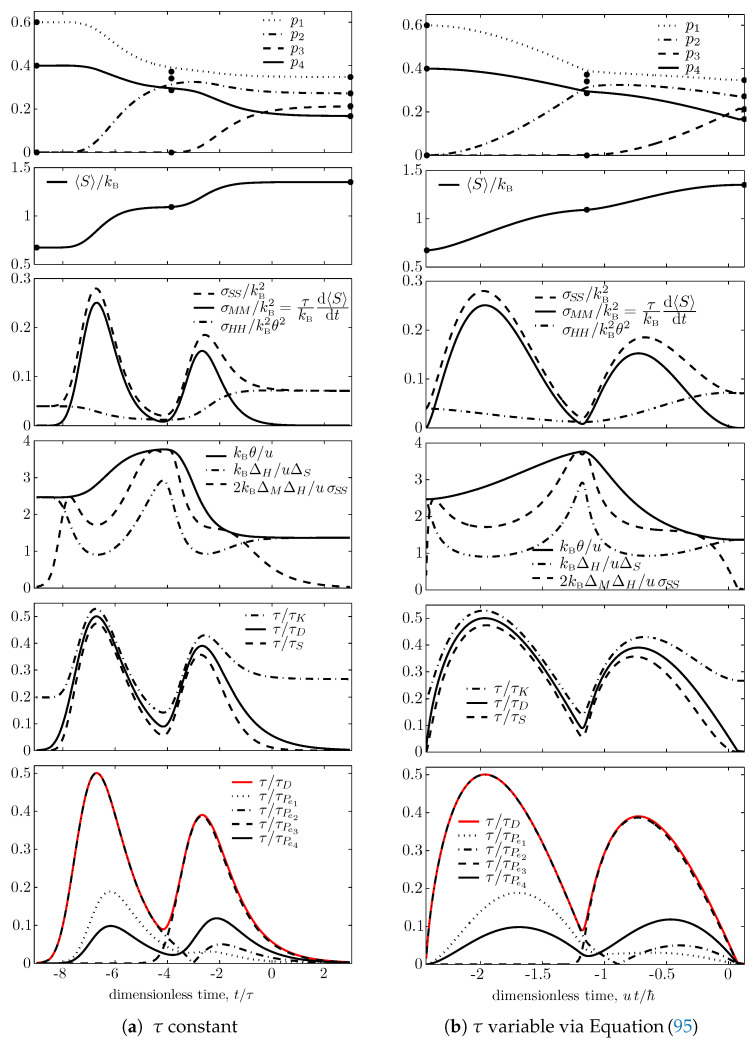
(color online) Time-dependent relaxation results obtained by integrating the steepest-entropy- ascent master Equation (89) for the four-level qudit with equally spaced energy levels, for two different choices of τ: (**a**) τ=const and (**b**) τ state-dependent according to Equation (95) and time non-dimensionalized by ℏ/u where *u* is the energy difference between the highest and lowest energy levels of the system. First row subfigures: Time evolution of the four occupation probabilities $p_{n}$. Second row: dimensionless entropy $\langle S\rangle /k_{B}$. Third row: rate of entropy change (proportional to $\sigma_{\;M\;M}$) compared with $\sigma_{\;S\;S}$ and $\sigma_{\;H\;H}/\theta^{2}$, to illustrate relation (73). Fourth row: generalized ‘nonequilibrium temperature’ θ (nondimensionalized by $u/k_{B}$) compared with $\Delta_{H}/\Delta_{S}$ and $2\Delta_{M}\Delta_{H}/\sigma_{\;S\;S}$ (also nondimensionalized) to illustrate relations (78) and (79). Fifth row: characteristic time of purely dissipative evolution $\tau_{D}$ (here proportional to the inverse of the square root of the rate of entropy generation, shown in the third row subplots) compared with $\tau_{S}$ and $\tau_{K}$ to illustrate relations (53) and (76). Sixth row: characteristic times of the four occupation probabilities $\tau_{P_{\;e_{\;n}}}$ compared with $\tau_{D}$ to illustrate relation (64).

**Figure 2:**
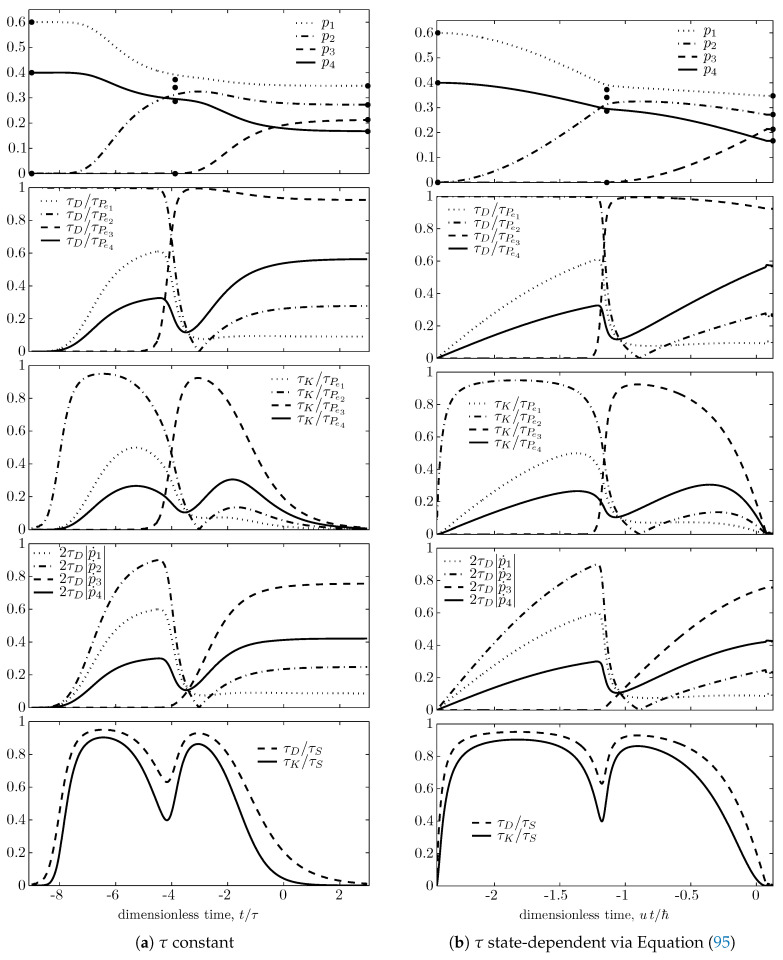
Time evolution of various other ratios of characteristic times for the same cases of Figure 1. First row subfigures: Time evolution of occupation probabilities $p_{n}$ (same as first row of Figure 1, repeated here for ease of comparison). Second row: ratios $\tau_{D}/\tau_{P_{\;e_{\;n}}}$ for each of the four occupation probabilities to illustrate again relation (64). Third row: $\tau_{K}/\tau_{P_{\;e_{\;n}}}$ to illustrate relation (86). Fourth row: $2\tau_{D}\;\left|{\dot{p}}_{n}\right|$ to illustrate relation (62). Fifth row: $\tau_{D}/\tau_{S}$ and $\tau_{K}/\tau_{S}$ to illustrate relations (53) and (84).

To obtain the plots in Figure 1 and Figure 2, that illustrate the main inequalities derived in this paper for a sample trajectory, we consider an initial state with cardinality equal to 4, with nonzero occupation probabilities only for the four energy levels $e_{1}=0$, $e_{2}=u/3$, $e_{3}=2u/3$, and $e_{4}=u$, and with mean energy 〈H〉=2u/5 (*u* is arbitrary, with units of energy). Moreover, as done in [94], we select an initial state ρ(0) at time t=0 such that the resulting trajectory ρ(t) passes in the neighborhood of the partially canonical nondissipative state $\rho_{\mathrm{nd}}^{\mathrm{ft}}$ that has nonzero occupation probabilities only for the three energy levels $e_{1}$, $e_{2}$, and $e_{4}$, and mean energy 〈H〉=2u/5 ($p_{\mathrm{nd}1}^{\mathrm{ft}}=0.3725$, $p_{\mathrm{nd}2}^{\mathrm{ft}}=0.3412$, $p_{\mathrm{nd}3}^{\mathrm{ft}}=0$, $p_{\mathrm{nd}4}^{\mathrm{ft}}=0.2863$, $\theta_{\mathrm{nd}}^{\mathrm{ft}}=3.796\;u/k_{B}$). As shown in Figure 1, during the first part of the trajectory, this nondissipative state appears as an attractor, an approximate or ‘false target’ equilibrium state; when the trajectory gets close to this state, the evolution slows down, the entropy generation drops almost to zero and the value of θ gets very close ($3.767\;u/k_{B}$) to that of $\theta_{\mathrm{nd}}^{\mathrm{ft}}$; however eventually the small, but nonzero initial occupation of level $e_{3}$ builds up and a new rapid rearrangement of the occupation probabilities takes place, and finally drives the system toward the maximal entropy state $\rho_{\mathrm{nd}}^{\mathrm{pe}}$ with energy 〈H〉=2u/5 and all four active levels occupied, with canonical (Gibbs) distribution $p_{\mathrm{nd}1}^{e}=0.3474$, $p_{\mathrm{nd}2}^{e}=0.2722$, $p_{\mathrm{nd}3}^{e}=0.2133$, $p_{\mathrm{nd}4}^{e}=0.1671$, and characterized by the equilibrium temperature $T_{e}=1.366\;u/k_{B}$.

The trajectory is computed by integrating Equation (89) numerically, both forward and backward in time, starting from the chosen initial state ρ(0), and assuming for Figure 1a and Figure 2a that the dissipation time τ is a constant, and for Figure 1b and Figure 2b that it is given by (26) with strict equality ($a_{\tau }=1$, $\tau_{D}=\tau_{U}$), i.e., assuming

(95) $$\begin{array}{ccc} \tau & = & \frac{\hbar /2}{k_{B}}\frac{\Delta_{M}}{\Delta_{H}}=\frac{\hbar /2}{k_{B}}\sqrt{\frac{\sigma_{\;S\;S}}{\sigma_{\;H\;H}}-\frac{1}{\theta^{2}}}\;, \end{array}$$

(96) $$\begin{array}{ccc} \sigma_{\;S\;S} & = & k_{B}^{2}\sum_{n}p_{n}{\left(\ln p_{n}\right)}^{2}-{\langle S\rangle }^{2}\;. \end{array}$$

The system of ordinary differential Equation (89) is highly nonlinear, especially when τ is assumed according to (95), nevertheless it is sufficiently well behaved to allow simple integration by means of a standard Runge–Kutta numerical scheme. Of course, we check that at all times −∞<t<∞ each $p_{n}$ remains nonnegative, $\sum_{n}p_{n}$ remains equal to unity, $\sum_{n}p_{n}e_{n}$ remains constant at the value 2u/5 fixed by the selected initial state, and the rate of change of 〈S〉 is always nonnegative.

In each Figure, the top subfigure shows for ease of comparison the plots of the four nonzero occupation probabilities as functions of dimensionless time: t/τ, in Figure 1a and Figure 2a; ut/ℏ, in Figure 1b and Figure 2b. The dots on the right represent the maximal entropy distribution, $p_{n}\left(+\infty \right)=p_{n}^{e}$; the dots at the left represent the lowest-entropy or ‘primordial’ distribution, $p_{n}\left(-\infty \right)=p_{\mathrm{nd}}^{\mathrm{ls}}$, which for the particular trajectory selected here, corresponds to a nondissipative state $\rho_{\mathrm{nd}}^{\mathrm{ls}}$ that has only two occupied energy levels, $e_{1}$ and $e_{4}$, with probabilities $p_{\mathrm{nd}1}^{\mathrm{ls}}=0.6$ and $p_{\mathrm{nd}4}^{\mathrm{ls}}=0.4$ (and temperature $T_{\mathrm{nd}}^{\mathrm{ls}}=2.466\;u/k_{B}$); in fact the four-level system has no lower entropy states ρ that commute with *H*, have energy 2u/5, and have zero occupation probabilities [94]. The dots in the middle represent the nondissipative state $\rho_{\mathrm{nd}}^{\mathrm{ft}}$ which appears as the false target state during the first part of the trajectory, plotted at the instant in time when the entropy of the time-varying trajectory is equal to the entropy of this distribution.

It is interesting to observe from Figure 1 (bottom subfigures) that during the early part of the trajectory, $\tau_{D}$ almost exactly coincides with $\tau_{P_{\;e_{2}}}$ while in the late part it almost exactly coincides with $\tau_{P_{\;e_{3}}}$, and the switch occurs when the trajectory slows down in the neighborhood of the ‘false target’ nondissipative state.

In Figure 1, the second subfigures show the time dependence of the dimensionless entropy $\langle S\rangle /k_{B}$; the third subfigures show its rate of change (proportional to $\sigma_{\;M\;M}$) and compares it with $\sigma_{\;S\;S}$ and $\sigma_{\;H\;H}/\theta^{2}$, to illustrate relation (73); the fourth show the time dependence of our generalized ‘nonequilibrium temperature’ θ (properly nondimensionalized) and compares it with $\Delta_{H}/\Delta_{S}$ and $2\Delta_{M}\Delta_{H}/\sigma_{\;S\;S}$ to illustrate relations (78) and (79); the fifth subfigures show the time dependence of $1/\tau_{D}$ (which here is proportional to the square root of the rate of entropy generation, third subfigures) and compares it with $1/\tau_{S}$ and $1/\tau_{K}$ to illustrate relations (53) and (76); the sixth subfigures show $1/\tau_{P_{\;e_{\;n}}}$ for each of the four occupation probabilities and compares them with $1/\tau_{D}$ to illustrate relation (64), which for this particular trajectory has the feature we just discussed.

In Figure 2, the second subfigures illustrate again relation (64) for each of the four observables $p_{n}=\langle P_{\;e_{\;n}}\rangle$; the third subfigures illustrate the time–entropy uncertainty relation (86) for the same observables; the fourth illustrate inequality (62); the fifth illustrate relations (53) and (84).

By comparing subfigures (a) and (b) in both Figure 1 and Figure 2, it is noted that most qualitative features remain the same when τ is changed from constant to the state-dependent functional defined by Equation (95), except for the almost singular behavior near the false target partially canonical nondissipative state, where $\Delta_{M}$ approaches zero and so does the dissipative time τ [Equation (95)]. The approach to final equilibrium in this case is not exponential in time as for τ= const. This puzzling behavior suggests that assumption (95) may hardly be physically sensible. However, as already noted after (24), it represents an interesting extreme behavior, i.e., the minimum dissipative time functional τ by which observables that commute with *H*, like the occupations $P_{\;e_{\;n}}$, never violate the usual time–energy uncertainty relations $\tau_{P_{\;e_{\;n}}}\Delta_{H}\geq \hbar /2$, even though their time dependence is not determined here by unitary dynamics but by purely dissipative dynamics. These usual time–energy uncertainty relations, $\tau_{P_{\;e_{\;n}}}\geq \tau_{U}$, are illustrated by the second row subfigures of Figure 2, because in this case $\tau_{U}=\tau_{D}$.

## 9. Conclusions

The Mandelstam–Tamm–Messiah time–energy uncertainty relation $\tau_{F}\Delta_{H}\geq \hbar /2$ provides a general lower bound to the characteristic times of change of all observables of a quantum system that can be expressed as linear functionals of the density operator ρ. This has been used to obtain estimates of rates of change and lifetimes of unstable states, without explicitly solving the time dependent evolution equation of the system. It may also be used as a general consistency check in measurements of time dependent phenomena. In this respect, the exact relation and inequalities (22) [that we derive for standard unitary dynamics based on the generalized Schrödinger inequality (14)] provide, for unitary evolution, a more general and sharper chain of consistency checks than the usual time–energy uncertainty relation.

The growing interest during the last three or four decades in quantum dynamical models of systems undergoing irreversible processes has been motivated by impressive technological advances in the manipulation of smaller and smaller systems, from the micrometer scale to the nanometer scale, and down to the single atom scale. The laws of thermodynamics, that fifty years ago were invariably understood as pertaining only to macroscopic phenomena, have gradually earned more attention and a central role in studies of mesoscopic phenomena first, and of microscopic and quantum phenomena more recently. In this paper we do not address the controversial issues currently under discussion about interpretational matters, nor do we attempt a reconstruction and review of the different views, detailed models and pioneering contributions that propelled during the past two decades this fascinating advance of thermodynamics towards the realm of few particle and single particle systems.

Motivated by this context and background, we derive various extensions of the usual time–energy uncertainty relations that may become useful in phenomenological studies of dissipative phenomena. We do so by focusing on a special but broad class of model evolution equations, that has been designed for the description of dissipative quantum phenomena and for satisfying a set of strict compatibility conditions with general thermodynamic principles. In this framework, we derive various forms of considerably precise time–energy and time–entropy uncertainty relations, and other interesting general inequalities, that should turn out to be useful at least as additional consistency checks in measurements of nonequilibrium states and time-dependent dissipative phenomena. To illustrate the qualitative features and the sharpness of the bounds provided by this set of inequalities, we show and discuss a numerical example obtained by integration (forward and backward in time) of the nonlinear evolution equation in the specific form introduced by this author for the description of steepest-entropy-ascent dynamics of an isolated system far from thermodynamic equilibrium.

## Appendix A. Reasons for Not Assuming a Kossakowski–Lindblad form of the Master Equation

With various motivations, fundamental or phenomenological, dissipative quantum dynamical models, i.e., evolution equations for the density operator ρ that do not conserve the functional −Tr(ρlnρ), are almost invariably based on the KLGS master equations. For example, in theories of open systems in contact with a heat bath, or subsystems of a composite system which as a whole evolves unitarily, a variety of successful model evolution equations for the reduced density operator of the system have the KLGS form [25,26,27,28,29,30,31,32,33]

(A1) $$\frac{d\rho }{dt}=-\frac{i}{\hbar }\left[H,\rho \right]+\frac{1}{2}\sum_{j}\left(2V_{j}^{†}\rho V_{j}-\left\{V_{j}^{†}V_{j},\rho \right\}\right)\;,$$

where the $V_{j}$’s are operators on H (each term within the summation, often written in the alternative form $\left[V_{j},\rho V_{j}^{†}\right]+\left[V_{j}\rho ,V_{j}^{†}\right]$, is obviously traceless). Evolution equations of this form are linear in the density operator ρ and preserve its hermiticity, nonnegativity and trace.

For example, in a number of successful models of dissipative quantum dynamics of open subsystems, operators $V_{j}$ are in general interpreted as creation and annihilation, or transition operators. For example, by choosing $V_{j}=c_{rs}\left|r\rangle \langle s\right|$, where $c_{rs}$ are complex scalars and |s〉 eigenvectors of the Hamiltonian operator *H*, and defining the transition probabilities $w_{rs}=c_{rs}c_{rs}^{*}$, Equation (A1) becomes

(A2) $$\frac{d\rho }{dt}=-\;\frac{i}{\hbar }\left[H,\;\rho \right]+\sum_{rs}w_{rs}\left(\left|s\rangle \langle \rho \rangle \langle s\right|-\;\frac{1}{2}\{|s\rangle \langle s|\;,\rho \}\right)\;,$$

or, equivalently, for the nm-th matrix element of ρ in the *H* representation,

(A3) $$\frac{d\rho_{nm}}{dt}=-\frac{i}{\hbar }\rho_{nm}\left(E_{n}-E_{m}\right)+\delta_{nm}\sum_{r}w_{nr}\rho_{rr}-\rho_{nm}\frac{1}{2}\sum_{r}\left(w_{rn}+w_{rm}\right)\;,$$

which, for the occupation probabilities $p_{n}=\rho_{nn}$, is the Pauli master equation

(A4) $$\frac{dp_{n}}{dt}=\sum_{r}w_{nr}p_{r}-p_{n}\sum_{r}w_{rn}\;.$$

Equation (A1) has also the intriguing feature of generating a completely positive dynamical map. However, Reference [125] argues quite clearly that the requirement of complete positivity of the reduced dynamics is too restrictive, as it is physically unnecessary to assure preservation of positivity of the density operator of the composite of any two noninteracting, uncorrelated systems.

Our objective here, instead, is to consider a class of model evolution equations applicable not only to open systems but also to closed isolated systems, capable of describing, simultaneously with the usual Hamiltonian unitary evolution, the natural tendency of any initial nonequilibrium state to relax towards canonical or partially-canonical thermodynamic equilibrium, i.e., capable of describing the irreversible tendency to evolve towards the highest entropy state compatible with the instantaneous mean values of the energy, the other constants of the motion, and possibly other constraints. To avoid the severe restrictions imposed by the linearity of the evolution equation, we open our attention to nonlinearity in the density operator ρ [84]. Therefore, it may at first appear natural to maintain the Kossakowski–Lindblad form (A1) and simply assume operators $V_{j}$ that are functions of ρ. This is true only in part for the evolution Equation (4) that we assume. Indeed, our hermitian operator $\Delta M\left(\rho \right)/k_{B}\tau$ can always be written as $-\sum_{j}V_{j}^{†}\left(\rho \right)V_{j}\left(\rho \right)$ and therefore our anticommutator term may be viewed as a generalization of the corresponding term in (A1).

However, in our Equations (1) and (4) we suppress the term corresponding to $\sum_{j}V_{j}^{†}\rho V_{j}$ in (A1). The reason for this suppression is the following. Due to the terms $V_{j}^{†}\rho V_{j}$, whenever the state operator ρ is singular, i.e., it has one or more zero eigenvalues, Equation (A1) implies that these zero eigenvalues may change at a finite rate. This can be seen clearly from (A4) by which $dp_{n}/dt$ is finite whenever there is a nonzero transition probability $w_{nr}$ from some other populated level ($p_{r}\neq 0$), regardless of whether $p_{n}$ is zero or not. When this occurs, for one instant in time the rate of entropy change is infinite, as seen clearly from the expression of the rate of entropy change implied by (A1),

(A5) $$\frac{d\langle S\rangle }{dt}=k_{B}\sum_{j}\mathrm{Tr}\left(V_{j}^{†}V_{j}\rho \ln\rho -V_{j}^{†}\rho V_{j}\ln\rho \right)=k_{B}\sum_{jrn}{\left(V_{j}\right)}_{nr}^{*}{\left(V_{j}\right)}_{nr}\left(\rho_{r}-\rho_{n}\right)\ln\rho_{r}\;,$$

where $\rho_{r}$ denotes the *r*-th eigenvalue of ρ and ${\left(V_{j}\right)}_{nr}$ the matrix elements of $V_{j}$ in the ρ representation.

We may argue that an infinite rate of entropy change can be tolerated, because it would last only for one instant in time. But the fact that zero eigenvalues of ρ in general could not survive, i.e., would not remain zero (or close to zero) for longer than one instant in time, is an unphysical feature, at least because it is in contrast with a wealth of successful models of physical systems in which great simplification is achieved by limiting our attention to a restricted subset of relevant eigenstates (forming a subspace of H that we call the effective Hilbert space of the system [81]). Such common practice *N*-level models yield extremely good results, that being reproducible, ought to be relatively robust with respect to including in the model other less relevant eigenstates. In fact, such added eigenstates, when initially unpopulated, are irrelevant if they remain unpopulated (or very little populated) for long times, so that neglecting their existence introduces very little error. The terms $V_{j}^{†}\rho V_{j}$, instead, would rapidly populate such irrelevant unpopulated eigenstates and void the validity of our so successful simple *N*-level models, unless we deliberately overlook this instability problem by highly ad-hoc assumption, e.g., by forcing the $V_{j}$’s to be such that ${\left(V_{j}\right)}_{nr}=0$ whenever either $\rho_{n}=0$ or $\rho_{r}=0$, in which case, however, we can no longer claim true linearity with respect to ρ.

To avoid the unphysical implications of this seldom recognized [81,94] problem of linear evolution equations of form (A1), we consider in this paper only equations of form (4). We do not exclude that it may be interesting to investigate also the behavior of equations that include nonlinear terms of the form $V_{j}^{†}\left(\rho \right)\;\rho \;V_{j}\left(\rho \right)$. However, at least when the system is strictly isolated, the operator-functions $V_{j}\left(\rho \right)$ should be such that ${\left(V_{j}\left(\rho \right)\right)}_{nr}=0$ whenever either $\rho_{n}=0$ or $\rho_{r}=0$.

Another important general physical reason why we exclude terms that generate nonzero rates of change of zero eigenvalues of ρ, is that if such terms are construed so as to conserve positivity in forward time, in general they cannot maintain positivity in backward time. The view implicitly assumed when Equation (A1) is adopted, is that the model is “mathematically irreversible” (a distinguishing feature if not a starting point of the theory of completely positive linear dynamical semigroups on which it is based), in the sense that neither uniqueness of solutions in forward time nor existence in backward time are required (and granted). Such mathematical irreversibility of the initial value problem, is often accepted, presented and justified as a natural counterpart of physical irreversibility. However, it is more related to the principle of causality than to physical irreversibility. The strongest form of the non-relativistic principle of causality—a keystone of traditional physical thought—requires that future states of a system should unfold deterministically from initial states along smooth unique trajectories in state domain defined for all times (future as well as past). Accepting mathematical irreversibility of the model dynamics implies giving up such causality requirement. The point is that such requirement is not strictly necessary to describe physical irreversibility, at least not if we are willing to give up linearity instead. The proof of this statement is our Equation (4) which, together with the additional assumptions made in Section 7 to describe relaxation within an isolated system, is mathematically reversible, in the sense that it features existence and uniqueness of well-defined solutions both in forward and backward time, and yet it does describe physically irreversible time evolutions, in the sense that the physical property described by the entropy functional $-k_{B}\mathrm{Tr}\left(\rho \ln\rho \right)$ is a strictly increasing function of time for all states except the very restricted subset defined by Equation (74), where it is time invariant.

## Appendix B. How Did Locally Steepest Entropy Ascent Come About?

The KLGS master equation emerges from a bottom-up phenomenological approach, whereby one considers a weakly interacting system+bath isolated composite evolving under the phenomenological assumption that the large number of degrees of freedom of the bath dilutes and destroys the correlations that build up under the standard unitary evolution due to the interaction term in the Hamiltonian, so that for the purpose of computing the evolution of the reduced density operator $\rho_{S}$ of the system, the overall state can be assumed to evolve through uncorrelated states, i.e., the system+bath density operator can at all times be written as $\rho =\rho_{S}⊗\rho_{B}$. So, we may say that the derivation is “bottom-up” because it starts from the fundamental unitary evolution of the composite system, but it is also “phenomenological” because the assumption of loss of correlations is an approximation that depends on the bath details and requires neglecting some terms during the partial tracing over the bath subspace.

By contrast, the locally steepest-entropy-ascent master equation was originally constructed (not derived) from a “top-down” approach, meant to see what modifications of standard quantum mechanics would be required if one wants to embed the second law of thermodynamics directly into the fundamental law of description. This heretic, but certainly intriguing and thought-provoking theoretical exercise, belongs to the early history of quantum thermodynamics and should not be forgotten. In the 70’s, the need for a quantum thermodynamics had been addressed boldly and explicitly only by Hatsopoulos and Gyftopoulos [63,64,65,66] and—with a very different approach that we do not review here—by Prigogine and the Brussels school of thermodynamics [126,127,128]. As an additional note pertaining to the history of thermodynamics, the course 2.47 J/22.58 J, listed in the MIT Bulletin of the academic year 1970-71 and taught jointly by George N. Hatsopoulos and Elias P. Gyftopoulos in the Spring of 1971, is the first official course entitled “Quantum Thermodynamics” that we are aware of.

An emphatic way to explain the (philosophical?) intuition of these pioneers of quantum thermodynamics, is the set of “what if” questions posed by the present author in a famous conference on the frontiers of nonequilibrium statistical physics held in Santa Fe in 1984 [129]: “what if entropy, rather than a statistical, information theoretic, macroscopic or phenomenological concept, were an intrinsic property of matter in the same sense as energy is universally understood to be an intrinsic property of matter? What if irreversibility were an intrinsic feature of the fundamental dynamical laws obeyed by all physical objects, macroscopic and microscopic, complex and simple, large and small? What if the second law of thermodynamics, in the hierarchy of physical laws, were at the same level as the fundamental laws of mechanics, such as the great conservation principles? Is it inevitable that the gap between mechanics and thermodynamics be bridged by resorting to the usual statistical, phenomenological, or information-theoretic reasoning, and by hinging on the hardly definable distinction between microscopic and macroscopic reality? Is it inevitable that irreversibility be explained by designing ad hoc mechanisms of coupling with some heat bath, reservoir or environment, and ad hoc mechanisms of loss of correlation? What if, instead, mechanics and thermodynamics were both special cases of a more general unified fundamental physical theory valid for all systems, including a single strictly isolated particle, such as a single isolated harmonic oscillator or a single isolated two-level spin system?”

In References [63,64,65,66] Hatsopoulos and Gyftopoulos showed that the only price we have to pay [130,131] to gain a possible positive answer to these questions is the reinterpretation of the physical meaning of the density operator, abandoning the standard (statistical mechanics and information theoretic) interpretation whereby it represents the epistemic ignorance of which particular pure state the system is ‘really’ in. Instead, the density operator acquires an ‘ontic’ status and represents the individual state of the isolated and uncorrelated atom (or particle or indivisible entity of the system’s model). Equivalently, in terms of ensembles, the density operator represents the measurement statistics from a homogeneous ensemble—homogeneous in the sense defined by von Neumann and discussed in References [57,82,87,88], i.e., such that no subsensemble can be identified which gives rise to different measurement statistics.

As a result of such ansatz, the severe restrictions imposed by linearity on the evolution equation become unnecessary, and we must open up our attention to evolution equations nonlinear in the density operator ρ. This is what prompted the search for a generalization of the time-dependent Schrödinger equation for pure density operators, to a broader fundamental kinematics in which not only every pure density operator, but also every non-pure density operator represents a real ontological object, the ‘true’ state of the system, which can be mixed even if the system is isolated and uncorrelated (no entanglement) from the rest of the universe. Among the desiderata [81] that drove the search for an extension, the most important was that the second law should emerge as a theorem of the equation of motion, which we considered the strongest way to enforce strong compatibility of a dynamical model (or law of motion or resource theory...) with thermodynamics.

For such purpose, the Hatsopoulos–Keenan statement of the second law is particularly suited, because it is directly linked with stability features of the equilibrium states of the dynamics. This somewhat still overlooked statement of the second law asserts ([86], p. 62) that for any well-defined (i.e., separable and uncorrelated) system, among the set of states that share the same values of the parameters of the Hamiltonian and the (mean) values of the energy and the amounts of constituents, there exists one and only one (conditionally [112,132]) stable equilibrium state, which turns out to be the one with the maximal entropy, often called the Gibbs state in the recent QT literature.

The Hatsopoulos–Keenan statement of the second law not only can be proved to entail the better known statements (Kelvin–Planck [86], p. 64; Clausius [86], p. 134; Caratheodory [86], p. 121), but—quite importantly for the current developments of quantum thermodynamics—it supports a rigorous operational definition of entropy as a general property of any uncorrelated (and unentangled) state of any well-separated system, valid not only for the stable equilibrium states of macroscopic systems but also for their nonequilibrium states (see Reference [133] and references therein) and providing a possible basis for its extension to systems with only few particles and quantum systems. Its extendability to correlated states of interacting or non-interacting systems is instead still the subject of intense debate, because the correlation entropy (often called mutual information), like the mean energy of interaction between the subsystems, is a well defined feature of the overall state of a composite system, but there is no unique way nor fundamental reason to allocate it among the subsystems and assign it to their local (reduced, marginal) states.

Using a similar operational definition, Hatsopoulos and Gyftopoulos in References [63,64,65,66] showed that the von Neumann entropy functional $-k_{B}\mathrm{Tr}\left(\rho \ln\rho \right)$ fulfills the definition of entropy. It was for such pioneering QT framework that the present author designed the nonlinear master equation [58,59,60] and shortly thereafter [61] proved it admits a steepest-entropy-ascent (SEA) variational formulation that embodies at the local microscopic level the principle of maximal entropy production [43,44,62,90,94,100,134].

Starting in the mid eighties, during times when quantum thermodynamics was considered interesting only by a handful of pioneers, the present author has addressed both the quantum-foundations and mathematical–physics communities [61,77,78,80,100,107,129,130,131,135,136,137,138] and the engineering-thermodynamics and non-equilibrium-thermodynamics communities [79,139,140,141,142,143,144] to raise awareness about the requirement that fundamental and phenomenological models of dissipative and irreversible processes must incorporate, i.e., must not violate, general thermodynamic principles.

More recently, the SEA principle has been shown to encompass all the major levels of description of nonequilibrium dynamics and irreversible processes [94], including the general modeling structure known as metriplectic dynamics [145,146,147] or GENERIC (see [35] and references therein), which even more recently has been shown to bear deep connections also with the mathematical theories of gradient flows [148,149,150] and large fluctuations [151,152].

In other words, the locally steepest-entropy-ascent model of far-non-equilibrium dissipative evolution in QT can be considered the most general precursor of all more recent and successful theories of nonequilibrium dynamical systems.

In several instances and different fields of application the LSEA approach has shown the ability to provide new nontrivial modeling capabilities not only in the realm of QT and related phenomenological resource theories (see, e.g., References [68,70,76]) but also in materials science [71,72,73,75] and transport theory [74].

Also the unified theory presented in References [63,64,65,66], if one puts aside the epistemic interpretation and considers it as an effective resource theory, represents in our view the pioneering precursor of many quantum thermodynamics results that have been re-derived in recent years (free energy versus available energy, energy versus entropy diagram, work element, adiabatic availability, etc.).
